# Exacerbated lung inflammation following secondary RSV exposure is CD4+ T cell-dependent and is not mitigated in infant BALB/c mice born to PreF-vaccinated dams

**DOI:** 10.3389/fimmu.2023.1206026

**Published:** 2023-08-14

**Authors:** Jessica L. Kosanovich, Katherine M. Eichinger, Madeline A. Lipp, Sonal V. Gidwani, Devarshi Brahmbhatt, Mark A. Yondola, Timothy N. Perkins, Kerry M. Empey

**Affiliations:** ^1^ Department of Pharmaceutical Sciences, University of Pittsburgh School of Pharmacy, University of Pittsburgh, Pittsburgh, PA, United States; ^2^ Calder Biosciences, New York, NY, United States; ^3^ Department of Pathology, University of Pittsburgh School of Medicine, Pittsburgh, PA, United States; ^4^ Department of Pharmacy and Therapeutics, University of Pittsburgh School of Pharmacy, University of Pittsburgh, Pittsburgh, PA, United States; ^5^ Center for Clinical Pharmaceutical Sciences, University of Pittsburgh School of Pharmacy, University of Pittsburgh, Pittsburgh, PA, United States; ^6^ Department of Immunology, University of Pittsburgh School of Medicine, University of Pittsburgh, Pittsburgh, PA, United States

**Keywords:** Respiratory syncytial virus, maternal immunization, type 2 inflammation, secondary RSV exposure, T cell-mediated pathology

## Abstract

Respiratory syncytial virus (RSV) is the leading cause of childhood hospitalizations due to bronchiolitis in children under 5 years of age. Moreover, severe RSV disease requiring hospitalization is associated with the subsequent development of wheezing and asthma. Due to the young age in which viral protection is needed and risk of vaccine enhanced disease following direct infant vaccination, current approaches aim to protect young children through maternal immunization strategies that boost neutralizing maternal antibody (matAb) levels. However, there is a scarcity of studies investigating the influence of maternal immunization on secondary immune responses to RSV in the offspring or whether the subsequent development of wheezing and asthma is mitigated. Toward this goal, our lab developed a murine model of maternal RSV vaccination and repeat RSV exposure to evaluate the changes in immune response and development of exacerbated lung inflammation on secondary RSV exposure in mice born to immunized dams. Despite complete protection following primary RSV exposure, offspring born to pre-fusion F (PreF)-vaccinated dams had exaggerated secondary ILC2 and Th2 responses, characterized by enhanced production of IL-4, IL-5, and IL-13. These enhanced type 2 cellular responses were associated with exaggerated airway eosinophilia and mucus hyperproduction upon re-exposure to RSV. Importantly, depletion of CD4^+^ T cells led to complete amelioration of the observed type 2 pathology on secondary RSV exposure. These unanticipated results highlight the need for additional studies that look beyond primary protection to better understand how maternal immunization shapes subsequent immune responses to repeat RSV exposure.

## 1 Introduction

Respiratory syncytial virus (RSV) remains the leading cause of childhood hospitalizations due to bronchiolitis in children under 5, leading to nearly 3.2 million hospitalizations and 60,000 in-hospital deaths each year (1). Furthermore, hospitalization as a result of RSV-associated bronchiolitis is strongly associated with the development of asthma and impaired lung function following repeat RSV exposure (2–4). Despite the morbidity that can result from severe infant RSV infection and subsequent disease sequelae, there is no licensed vaccine currently available for the prevention of RSV in young children. Because hospitalization for severe RSV-mediated disease peaks between 6 weeks and 6 months of age, when infants rely heavily on maternal antibody (matAb) for protection, current RSV vaccination strategies are largely focused on boosting maternally derived RSV-neutralizing antibody (5, 6). High levels of RSV-specific matAbs have been correlated with protection from severe early-life RSV disease and hospitalization (7–9). As such, maternal RSV vaccination has emerged as a promising approach and several maternal vaccine candidates have quickly progressed to late-stage clinical trials (NTC04605159; NTC04424316) (10).

Previous work in our lab has demonstrated that offspring challenged with RSV in the presence of highly neutralizing matAbs are not only fully protected from viral infection but have significant alterations in their immune response to RSV. Weanlings with high levels of neutralizing matAb had reduced eosinophilic infiltrate in their airways with a concomitant decrease in CD4^+^ and CD8^+^ T cell responses, which jointly reduced airway inflammation (11). In a similar model of maternal RSV immunization using cotton rats, Blanco et al. demonstrated that high titers of neutralizing matAb correlated with complete protection from viral challenge while also reducing the expression of the inflammatory cytokines, IFNγ and IL-6, and associated lung pathology (12). Collectively, these studies demonstrate that anti-RSV immunity in infants is influenced by the presence of highly neutralizing matAb. Yet, the effect these altered early-life immune responses have on long-lived immunity to RSV remains largely unknown.

Prior research has shown that early-life immune responses to RSV infection critically impacts airway responses in adulthood (13–15). Results from these studies have demonstrated that mice first exposed to RSV as neonates have increased airway inflammation, characterized by eosinophilia, increased IL-13 production, and associated mucus hyperproduction upon secondary RSV infection or exposure to allergen in adulthood (14, 15). It is generally assumed that if neonatal RSV infection can be avoided via matAb neutralization, these maladaptive early-life immune responses will be mitigated. However, the effect of matAb on the development of immunity is well-appreciated in models of direct infant immunization, and yet, there is a dearth of data regarding the influence of matAb on active immunity following natural antigen re-exposure (16, 17). Toward this goal, our lab developed a murine model of maternal RSV vaccination and repeat RSV exposure to evaluate the changes in immune response that influence the safety and efficacy of maternal RSV immunization following RSV re-exposure.

## 2 Materials and methods

### 2.1 Maternal immunization, intranasal RSV infections, and treatments

Animal studies were carried out in accordance with the University of Pittsburgh’s IACUC guidelines for the use and care of laboratory animals. Female Balb/cJ mice (7-8 weeks of age; The Jackson Laboratory, Bar Harbor, ME) were primed one week prior to breeding via intramuscular (i.m.) hind-limb injection with 50μL of PBS alone or DS-Cav1 (10μg per mouse; a generous gift from Jason McLellan and supplied by Calder Biosciences, Brooklyn, NY) formulated with Alum (100μg per mouse; Alhydrogel, Invivogen). One week later, mice were bred, as previously described (18) and in the second week of gestation (3 weeks post-prime), mice were boosted i.m. with their respective vaccine. Offspring born to PBS-vaccinated dams are referred to as mVeh, while those born to DS-Cav1+Alum-vaccinated dams are labeled as mAlum. It has been previously demonstrated in a model of maternal formalin-inactivated RSV immunization, that vaccine-mediated Th2-skewing in dams is not transferred to offspring (19). Based on this, an alum-only group (Th2-skewing) was not included in these studies.

For primary RSV infections, mice were intranasally (i.n.) infected with RSV L19 (provided by Dr. Martin Moore) at an infectious dose of 5x10^5^ plaque forming units (PFU) per gram of body weight at post-natal day (PND) 5-6 under isoflurane anesthesia, as previously described (18). For secondary RSV infections, maternally vaccinated offspring were aged to adulthood (~9 weeks of age) before i.n. infection with 5x10^5^ PFU/gm RSV L19 under isoflurane anesthesia. At 4- and 8-days post-exposure, mice were culled using 100% isoflurance and cervical dislocation. RSV L19 was propagated and viral titers quantified as previously described (20).

For CD4^+^ T cell depletion, mice were treated with 200μg of α-CD4 antibody (α-CD4; clone GK1.5; BioLegend) via intraperitoneal (i.p.) injection every 2 days starting at day -1 prior to RSV exposure and through sacrifice at 4- or 8-days post-exposure, as previously described (21). Control mice (IgG) were administered matching doses of control IgG2b antibody (IgG; BioLegend).

### 2.2 RSV-neutralizing antibody – Renilla Luciferase RSV reporter assay

Neutralizing antibody titers were determined by an assay established in our laboratory and previously reported (11, 22–24). In infants, pre-challenge blood was collected via terminal bleed from non-exposed infants at PND5-6. In adult offspring, pre-challenge blood was collected via submandibular bleed immediately prior to RSV exposure and separated using Gel-Z serum separator tubes (Sarstedt). Serum was stored at -80°C until heat-inactivation (56°C for 30 minutes) and Renilla Luciferase RSV Reporter Assay was performed (11, 22–24). Briefly, heat-inactivated serum was serially diluted in phenol-free MEM supplemented with 5% FBS and Pen/Strep before incubation in 96-well plate format with 100 PFU/well RSV L19-Renilla Luciferase virus (a generous gift from Martin Moore) for 2 hours at 37°C/5% CO_2_. After incubation, HEp-2 cells were trypsinized and a total of 2.5x10^4^ cells were added to 25μL FBS+Pen/Strep-containing phenol-free MEM and incubated for 64-66 hours at 37°C/5% CO_2_. After incubation, luciferase readouts were obtained using the Renilla-Glo Luciferase Assay system (Promega), according to manufacturer’s instructions. After incubation, luciferase activity was measured using a Novostar plate reader. The RSV luciferase assay generates a sigmoidal luminescence readout from which the midpoint (IC50) is calculated by nonlinear regression in a manner previously described for fluorescence and luminescence-based assays (25–27). Serum dilutions are used to generate a full sigmoidal-shaped luminescence curve for the vaccinated animals and control samples are diluted equivalently to allow for cross-comparison. The limit of blank (cells only control) was previously determined for this assay to be 1826 RLU and the average of the virus-only control lanes included on each plate in the assay was 60,630 RLU. Since no Veh control samples achieved a signal less than 80% of the virus-only control even at their highest concentration, no IC50 could be determined for these samples and the lowest serum dilution used in the assay is reported as the neutralization titer, in this case 1:100 (marked by a dashed line). All plates were run in duplicate and averaged.

### 2.3 RSV-specific IgG subtype assays

Co-star 96-well, high binding ELISA plates were coated with DS-Cav1 at a concentration of 5μg/mL overnight at 4°C. Each plate included standards of either mouse IgG1 or IgG2a (Invitrogen) at 10μg/mL and 2μg/mL, respectively in a 2-fold dilution series. Plates were then washed with PBS and blocked for 1 hour at 37°C with 1% BSA in PBS. Heat-inactivated serum samples were diluted 1:500 in 1% BSA in PBS for the first well, and then 3-fold serially diluted a total of 3 times. Serum was incubated on the plates for 1 hour at 25°C, followed by 3 washes with PBS 0.05% Tween-20, and secondary antibody incubation with anti-IgG1 or anti-IgG2a (isotype specific, BD Pharmingen) at a 1:10,000 dilution for 30 minutes at 25°C in 1% BSA. 1-step TMB (Thermo Scientific) was used to develop the plates and the reaction was quenched by the addition of 4N H_2_SO_4_. Plates were read at 450 nm using a Novostar plate reader. Data analysis was performed in Excel and data points were interpolated from the linear region of the standards on each individual plate.

### 2.4 Cell preparation, stimulation, and flow cytometry

Bronchoalveolar lavage (BAL) and right lung lobes were collected, processed, and enumerated, as previously described (28). For innate immune cell stimulation, BAL cells were incubated for 3 hours in 10% RPMI supplemented with Brefeldin A (1:1000, eBioscience). For ILC2 stimulation, lung homogenate was stimulated with PMA (30 ng/mL, Sigma Aldrich), ionomycin (500 ng/mL, Sigma Aldrich), and Brefeldin A (1:1000) in 10% RPMI at 37°C for 3 hours. For T cell stimulation, BAL cells were stimulated with plate-bound CD3 (5μg/mL, Biolegend) in 10% RPMI supplemented with CD28 (2μg/mL) and incubated at 37°C overnight. After overnight stimulation with CD3/CD28, lung homogenate underwent a secondary stimulation with PMA (10ng/mL), ionomycin (1μg/mL), and Brefeldin A (1:1000) for 2 hours. Following stimulation, BAL and lung cells were surface stained, then fixed and permeabilized using BD Cytofix/Cytoperm™ kit (BD Biosciences) prior to intracellular cytokine staining. For innate cell identification (**Supplementary Figure 1**), BAL was surface stained with CD16/32 (Fc block; 2.4G2), LIVE/DEAD™ Fixable Blue Dead Cell Stain Kit, CD11c (N418), CD11b (M1/70), SiglecF (E50-2440), F4/80 (T45-2342), CD200R (OX-110), and Ly6G (1A8) and intracellularly stained for CD206 (C0698C2), TNFα (MP6-XT22), and IL-10 (JES5-16E3). For identification of ILC2s (**Supplementary Figure 2**), lung cells were surface stained with CD16/32 (Fc block; 2.4G2), LIVE/DEAD™ Fixable Blue Dead Cell Stain Kit, Lineage Cocktail (CD3 (17A2), Ly6G/Ly6C (RB6-8C5), CD11b (M1/70), CD45R (RA3-6B2), TER-119 (Ter-119)), CD49b (DX5), CD45 (30-F11), ST2 (DIH9), ICOS, (C398.4A), IL-25R (6B7) and CD25 (PC61) and intracellularly stained for IL-5 (TRFK5) and IL-13 (ebio13A). For T cell identification (**Supplementary Figure 3**), BAL was surface stained with CD16/32 (Fc block; 2.4G2), LIVE/DEAD™ Fixable Blue Dead Cell Stain Kit, TCRβ (H57-597), CD8 (53-6.7), CD4 (GK1.5), CD44 (IM7), and CD25 (PC61) and intracellularly stained with TCRβ (H57-597), CD4 (54-6.7), IFNγ (XMG1.2), IL-4 (11B11), IL-5 (TRFK5), IL-13 (ebio13A), and Granzyme B (QA16A02). Samples were run on a BD Fortessa or Cytek Aurora managed by the United Flow Core of the University of Pittsburgh. Data was analyzed using FlowJo V10 software (FLOWJO, LLC, OR).

### 2.5 Histology

Left lungs were gravity-filled with 10% formalin at 4- and 8-days post RSV exposure, as previously described (29). Lungs were paraffin-embedded, processed and stained with hematoxylin and eosin or Periodic Acid-Schiff (PAS) at the McGowan Institute for Regenerative Medicine (University of Pittsburgh, PA). Lung inflammation and mucus hypersecretion were quantified by pathologists blinded to treatment groups, as previously described (18, 30). To assess lung inflammation histologically, H&E stained lung sections were blinded and then semi-quantitatively scored. For each specimen, all broncho-vascular bundles were given a score indicating the relative intensity of inflammation (i.e. number of inflammatory cells) in the affected area. For scoring, 0 = no inflammation, 1 = mild inflammation, 2 = moderate inflammation, and 3 = severe inflammation. Scores were then averaged to give an overall score for each specimen. For PAS staining of mucus, all airways were scored for the percentage of airway staining PAS+ (bright pink) in each tissue section according to the following scale: 0 =no PAS+ cells; 1 = 1-25% PAS+ cells; 2 = 26-50% PAS+ cells; 3 = 51-75% PAS+ cells; 4 = 76-100% PAS+ cells. PAS severity is reported as: none (percentage of airways with a score of 0), mild (percentage of airways with a score of 1 and 2) or severe (percentage of airways with a score of 3 and 4).

### 2.6 Statistical analysis

Statistical analyses were performed with GraphPad Prism 9 software (GraphPad Software, La Jolla, CA). Results are displayed as the mean ± SEM. For most analyses, data are compared using an unpaired t-test. Neutralizing antibody data was analyzed by nonlinear regression to obtain IC50 values, which were compared between groups using an unpaired t-test. For nonparametric ratio data, a Wilcoxon signed rank test was performed to compare the median of each group to a theoretical median of 1. For multiple comparisons, a one-way analysis of variance (ANOVA) with Tukey’s multiple comparisons test was performed to evaluate statistical significance between groups. For experiments where multiple groups were compared over multiple timepoints, treatments, or RSV exposures, a two-way ANOVA with Sidak’s multiple comparisons test was used to evaluate statistical significance between groups. p values ≤0.05 were considered significant.

## 3 Results

### 3.1 Offspring born to PreF+Alum-immunized dams are protected following primary and secondary RSV exposure

**Figure 1:**
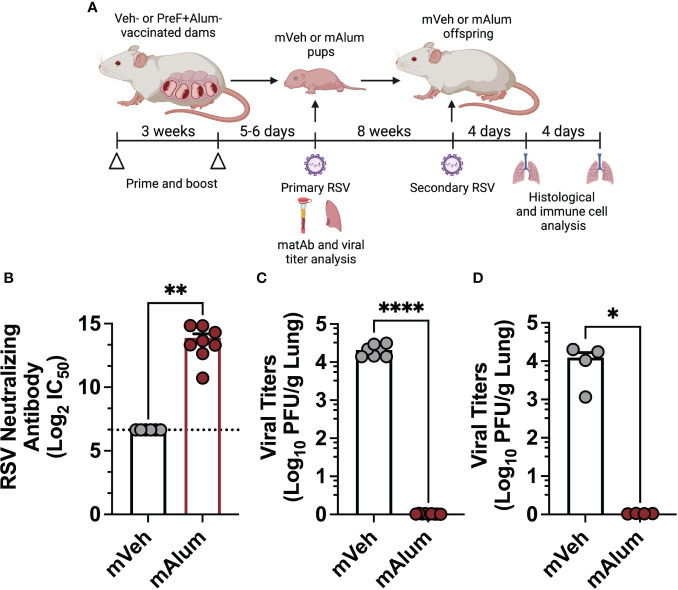
Offspring born to PreF+Alum-vaccinated dams are protected from primary and secondary RSV exposure. Pregnant Balb/c mice completed a 2-dose vaccination series of PBS (mVeh) or preF+Alum 1 week prior to parturition. At PND5-6, a cohort of mVeh and mAlum pups were culled for pre-challenge serum antibody analysis while a second cohort of pups were intranasally exposed to 5x10^5^ PFU/gm RSV L19 and culled a 4-days post-exposure for viral titer analysis. A third cohort of mVeh and mAlum offspring were aged to adulthood, re-exposed to RSV L19 at 9 weeks old (PND63) and culled for sample collection at 4- and 8-days post-exposure **(A)**. Pre-challenge serum from mVeh and mAlum pups was analyzed for RSV neutralizing antibody as described in the methods; since no dilution of Veh control samples achieved a signal less than 80% of the limit of blank-corrected virus-only control, no IC50 could be determined for these samples and the lowest serum dilution used in the assay is reported as the neutralization titer, in this case 1:100 (marked by a dashed line) **(B)**. Left lungs were harvested from pups **(C)** and aged adults for viral titer analysis **(D)**. Data are represented as mean ± SEM (n=4-9 mice per group). Statistical significance was calculated using an unpaired t-test between mVeh and mAlum offspring. *p ≤ 0.05, **p ≤ 0.01 and ****p ≤ 0.0001.

To evaluate the effect of high levels of RSV-neutralizing maternal antibody (matAb) on the immune response to secondary RSV exposure, our previously established model of maternal vaccination and infant RSV infection was adapted to include a second RSV exposure in adulthood (11). Pups born to dams immunized with PBS (mVeh) or PreF+Alum (mAlum) remained unchallenged or received an intranasal RSV L19 challenge at post-natal day (PND) 6, as previously described (18). Serum was collected from unchallenged offspring at PND6 to measure RSV-neutralizing antibody levels. RSV viral lung titers were measured in a cohort of RSV-challenged pups at 4 days post-exposure (dpe); the remaining cohort of RSV challenged pups were aged to adulthood. At 9 weeks old, an age at which RSV-neutralizing matAb is markedly reduced (**Supplementary Figure 4A**), mVeh and mAlum offspring were then re-challenged with RSV L19 and lung histology and immune cell analysis were performed at 4- and 8-days post-secondary exposure (dpse) (**Figure 1A**). As expected, mVeh pups had undetectable levels of RSV-neutralizing antibody at time of primary intranasal RSV L19 challenge (**Figure 1B**). In comparison, mAlum pups had high levels of RSV-neutralizing antibody (**Figure 1B**), which was associated with undetectable levels of RSV replication at 4dpe (**Figure 1C**). Unlike mVeh offspring, mAlum offspring were also protected from detectable replicating RSV following secondary challenge (**Figure 1D**). mAlum offspring receiving a primary RSV infection at 9 weeks of age (PND63) have detectable titers of RSV-neutralizing matAb, but levels were insufficient to completely protect from replicating virus (**Supplementary Figures 4A, B**). Analysis of the RSV-specific IgG1/IgG2a ratio prior to primary RSV exposure was above 1 in the mAlum pups and remained elevated through PND63 (prior to secondary RSV exposure), suggesting a Th2-skewed response in the dams (**Supplementary Figure 4C**). These data confirm that offspring born to maternally immunized dams are completely protected against replicating RSV following primary and secondary challenge.

### 3.2 mAlum offspring have a Th2-skewed CD4^+^ T cell profile with reduced cytotoxic CD8^+^ T cell activity

**Figure 2:**
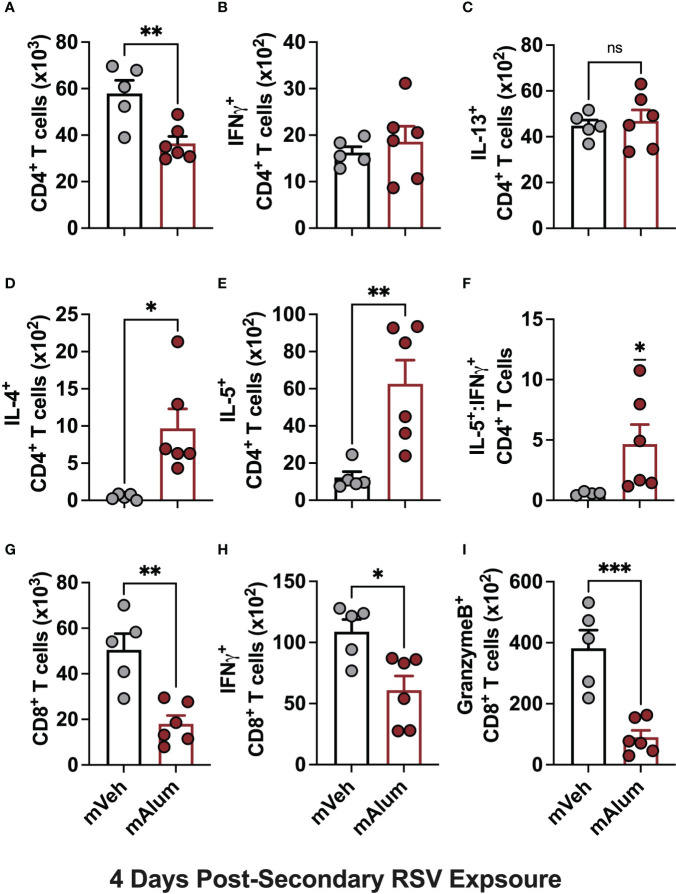
mAlum offspring have a dominant Th2 phenotype with reduced cytotoxic CD8^+^ T cell activity. mVeh and mAlum offspring were treated as described in **Figure 1A**. At 4dpse, total BAL CD4^+^ T cells **(A)**, as well as intracellular production of IFNγ, **(B)**, IL-13 **(C)**, IL-4 **(D)**, and IL-5 **(E)** by CD4^+^ T cells were quantified. From these totals, the ratio of IL-5:IFNγ CD4^+^ T cells was calculated **(F)**. At the same timepoint, total BAL CD8^+^ T cells **(G)**, as well as those producing IFNγ **(H)**, and GranzymeB **(I)** were quantified. Data are represented as mean ± SEM (n=5-6 mice per group). Statistical significance was calculated using an unpaired t-test **(A-E, G-I)** or Wilcoxon signed-rank test **(F)**. ns – non-significant, *p ≤ 0.05, **p ≤ 0.01 and ***p ≤ 0.001.

Several studies have shown that primary RSV exposure during infancy, but not in adulthood, leads to exaggerated Th2 responses upon RSV re-infection (13–15). To assess whether presence of matAb during primary infant RSV challenge alters the CD4^+^ Th2 bias that occurs on repeat RSV exposure, cells of the bronchioalveolar lavage (BAL) fluid were stained for flow cytometric analysis at 4dpse. Total CD4^+^ T cells were significantly reduced in mAlum compared to mVeh offspring (**Figure 2A**). No significant differences were observed in IFNγ^+^ (**Figure 2B**) or IL-13^+^ (**Figure 2C**) CD4^+^ T cells between groups. However, IL-4^+^ (**Figure 2D**) and IL-5^+^ (**Figure 2E**) CD4^+^ T cells were increased in mAlum offspring, resulting in a significantly higher IL-5^+^:IFNγ^+^ CD4^+^ T cell ratio compared to mVeh offspring (**Figure 2F**). Together, these data identify a heavily Th2-skewed CD4^+^ T cell phenotype in mAlum versus mVeh offspring following secondary RSV exposure. It also suggests that the presence of RSV-neutralizing matAb and protection against RSV replication at primary infant challenge does not mitigate the establishment and activation of Th2 CD4^+^ T cells upon RSV re-challenge.

Activation of CD8^+^ T cells, which is critical for RSV immunity (31, 32), was also assessed in mVeh and mAlum offspring following RSV re-challenge. At 4dpse, total CD8^+^ T cells (**Figure 2G**), along with those producing IFNγ (**Figure 2H**) and GranzymeB (**Figure 2I**) were significantly reduced in mAlum vs. mVeh offspring, which is consistent with previous work demonstrating the matAb reduces secondary CD8^+^ T cell responses (33). These data collectively show that mAlum offspring have a heavily Th2-skewed CD4^+^ T cell phenotype in combination with reduced cytotoxic CD8^+^ T cells upon secondary RSV exposure.

### 3.3 IL-5- and IL-13-producing ILC2s are significantly elevated in the lungs of mAlum offspring

**Figure 3:**
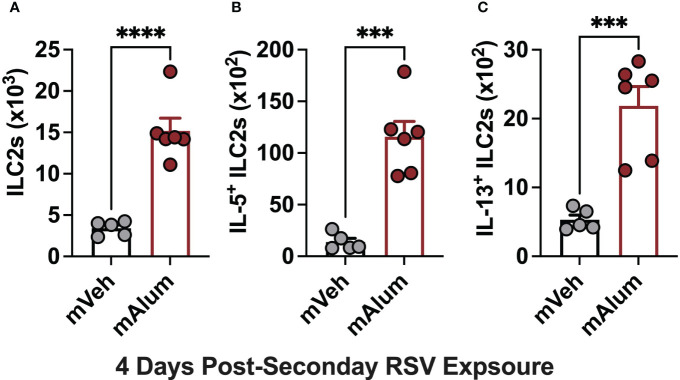
Enhanced ILC2 activity in mAlum offspring following secondary RSV exposure. mVeh and mAlum offspring were generated and exposed to RSV as described in **Figure 1**. Total lung ILC2s **(A)**, along with those producing IL-5 **(B)** and IL-13 **(C)** were quantified at 4dpse. Data are represented as mean ± SEM (n=5-6 mice per group). Statistical significance was calculated using an unpaired t-test. ***p ≤ 0.001 and ****p ≤ 0.0001.

There is a growing body of evidence implicating ILC2s as a significant and potent source of type-2 cytokines capable of driving RSV-mediated immunopathology (34, 35). Importantly, neonatally primed ILC2s are associated with exacerbated airway inflammation following RSV re-infection (36). Therefore, to determine if the presence of matAb during primary neonatal RSV exposure attenuates secondary ILC2 responses, lung ILC2s from mVeh and mAlum offspring were assessed via flow cytometry following secondary RSV exposure. Total ILC2s (**Figure 3A**) and those producing IL-5 (**Figure 3B**) and IL-13 (**Figure 3C**) were significantly increased in the lungs of mAlum compared to mVeh offspring at 4dpse, despite no detectable viral replication at the same timepoint (**Figure 1D**). Together, these data suggest that the presence of matAb during primary RSV exposure is associated with exaggerated secondary ILC2 responses.

### 3.4 ILC2s with a hyperresponsive phenotype are enriched in lungs of mAlum offspring

**Figure 4:**
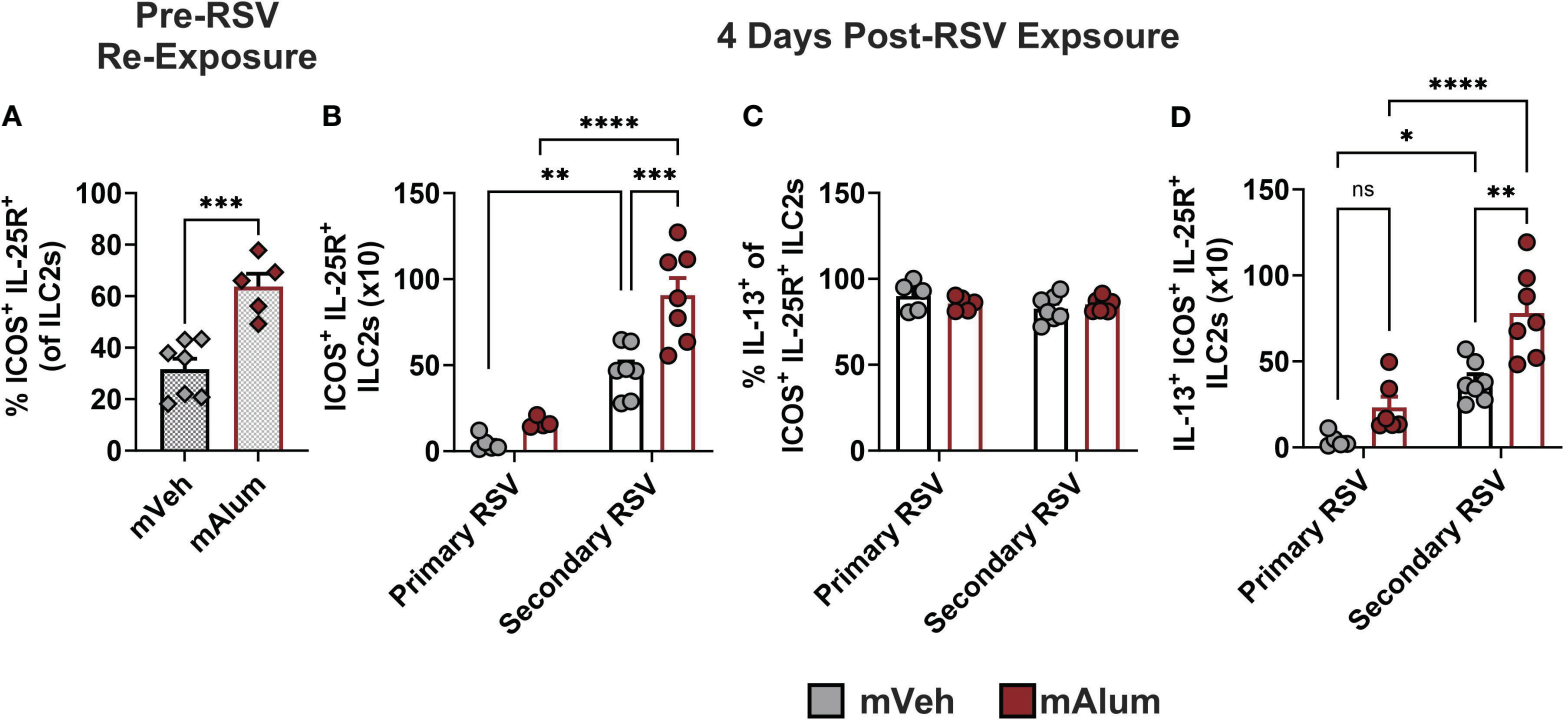
ILC2s with hyperresponsive phenotype are enriched in lungs of mAlum offspring. The frequency of hyperresponsive ILC2s, characterized by surface expression of ICOS and IL-25R, was assessed in the lungs of mVeh and mAlum offspring prior to secondary RSV exposure **(A)**. Total ICOS^+^ IL-25R^+^ ILC2s **(B)**, the frequency of IL-13-producing ICOS^+^ IL-25R^+^ ILC2s **(C)** and IL-13^+^ ICOS^+^ IL-25R^+^ ILC2s **(D)** were quantified at 4 days post-exposure in the lungs of adult mVeh and mAlum offspring following primary (1°) and secondary (2°) RSV exposure. Data are represented as mean ± SEM (n=5-7 mice per group). Statistical significance was calculated using an unpaired t-test **(A)** or two-way ANOVA with Sidak’s multiple comparison test **(B-D)** between groups. ns – nonsignificant, *p ≤ 0.05 **p ≤ 0.01, ***p ≤ 0.001 and ****p ≤ 0.0001.

A hyperresponsive, memory-like ILC2 population, characterized by IL-25R and ICOS expression, has been described in multiple models of allergic lung inflammation (37, 38). After initial activation and expansion, a subset of ILC2s undergo contraction and remain long-lived in the lungs (37). These hyperresponsive ILC2s (hILC2s) produce exaggerated amounts of IL-5 and IL-13 in response to subthreshold levels of the initial, sensitizing signal or unrelated antigens, akin to the trained immunity described in other innate immune cells (38). Given the exaggerated ILC2 responses observed following RSV re-challenge (**Figures 3A–C**), we hypothesized that the hyperresponsive ICOS^+^ IL-25R^+^ ILC2 population was increased following secondary RSV exposure in the lungs of mAlum vs mVeh offspring. Prior to secondary RSV exposure, a significantly higher frequency of ILC2s resident in the lungs of mAlum offspring had dual expression of ICOS and IL-25R compared to mVeh offspring (**Figure 4A**). This suggests that mAlum offspring have higher frequencies of hILC2s present in their lungs poised for re-activation.

Upon secondary RSV exposure, hILC2s were significantly increased in mAlum offspring compared to mVeh offspring (**Figure 4B**; Secondary RSV). To confirm that this hILC2 response on secondary challenge could not be attributed to a primary adult ILC2 response or lingering matAb in the offspring, adult mVeh and mAlum offspring were exposed to RSV for the first time at 9 weeks of age, the same age as offspring receiving a secondary exposure. Primary ILC2 responses in mVeh and mAlum offspring were 5-10x lower than that observed following secondary RSV exposure (**Figure 4B**; Primary RSV). Moreover, mAlum compared to mVeh offspring had significantly more hILC2s on secondary RSV exposure, suggesting that the presence of matAb on primary infection does not mitigate the exaggerated ILC2 response on repeat challenge. Though no significant difference was observed in IL-13-producing hILC2s between mVeh vs. mAlum offspring during primary adult RSV infection (**Figure 4C**), both mVeh and mAlum offspring had significantly more IL-13^+^ hILC2s upon RSV re-exposure (**Figure 4C**). Nearly all ILC2s expressed IL-13 in both groups at primary and secondary RSV challenge (**Figure 4C**), however, RSV re-challenge resulted in significantly more IL-13^+^ hILC2s in mAlum offspring compared to mVeh offspring (**Figure 4D**). Taken together, these results suggest that the presence of preF-neutralizing matAb during primary RSV infection increases the number of lung-resident ICOS^+^ IL-25R^+^ hILC2s, allowing for rapid and exaggerated type-2 responses to RSV re-exposure.

### 3.5 mVeh and mAlum offspring have severe airway mucus metaplasia following RSV re-exposure

Several studies have shown that neonatal RSV infection predisposes adult mice to IL-13-dependent mucus hyperproduction and enhanced airway hyperresponsiveness following RSV re-infection (14, 15). Enhanced airway responses observed on secondary infection were shown to require active lung infection during initial RSV exposure (14). Although mAlum offspring were completely protected from active primary and secondary RSV lung infection (**Figures 1C, D** **)**, the heavily skewed Th2 phenotype (**Figure 2**) combined with the hyperactivation of ILC2s (**Figures 3**, **4**) suggests that mAlum offspring may not be protected from the immunopathology associated with secondary RSV challenge. To this end, airway inflammation and mucus metaplasia were assessed in the lungs of mVeh and mAlum offspring at 4dpse. Airways were first lavaged and inflammatory cells were quantified by flow cytometry. Analysis of innate cells in the BAL revealed that mAlum offspring had significantly more monocytes (**Supplementary Figure 5A**) and eosinophils (**Supplementary Figure 5D**) in their airways compared to mVeh offspring. The number of alveolar macrophages (**Supplementary Figure 5E**) and neutrophils (**Supplementary Figure 5H**) were similar between groups. Interestingly, the alveolar macrophage and monocyte populations had a predominant pro-inflammatory phenotype in mAlum offspring, evidenced by their increase in TNFα production and a parallel decrease in IL-10 (**Supplementary Figures 5B, C, F-G**), a phenotype implicated in orchestrating and exacerbating pulmonary inflammation (15). After the lungs were lavaged they were harvested for histopathology to further assess inflammation of the bronchovascular bundles. mVeh and mAlum offspring had similar frequencies of bronchovascular bundles with inflammation (**Supplementary Figures 6A-C**), with each having a similar distribution of severity (**Supplementary Figures 6D**). Together, these data indicate that mAlum compared to mVeh offspring had an increase in inflammatory cells in the alveolar space, but no differences in inflammation were observed within the bronchovascular bundles.

**Figure 5:**
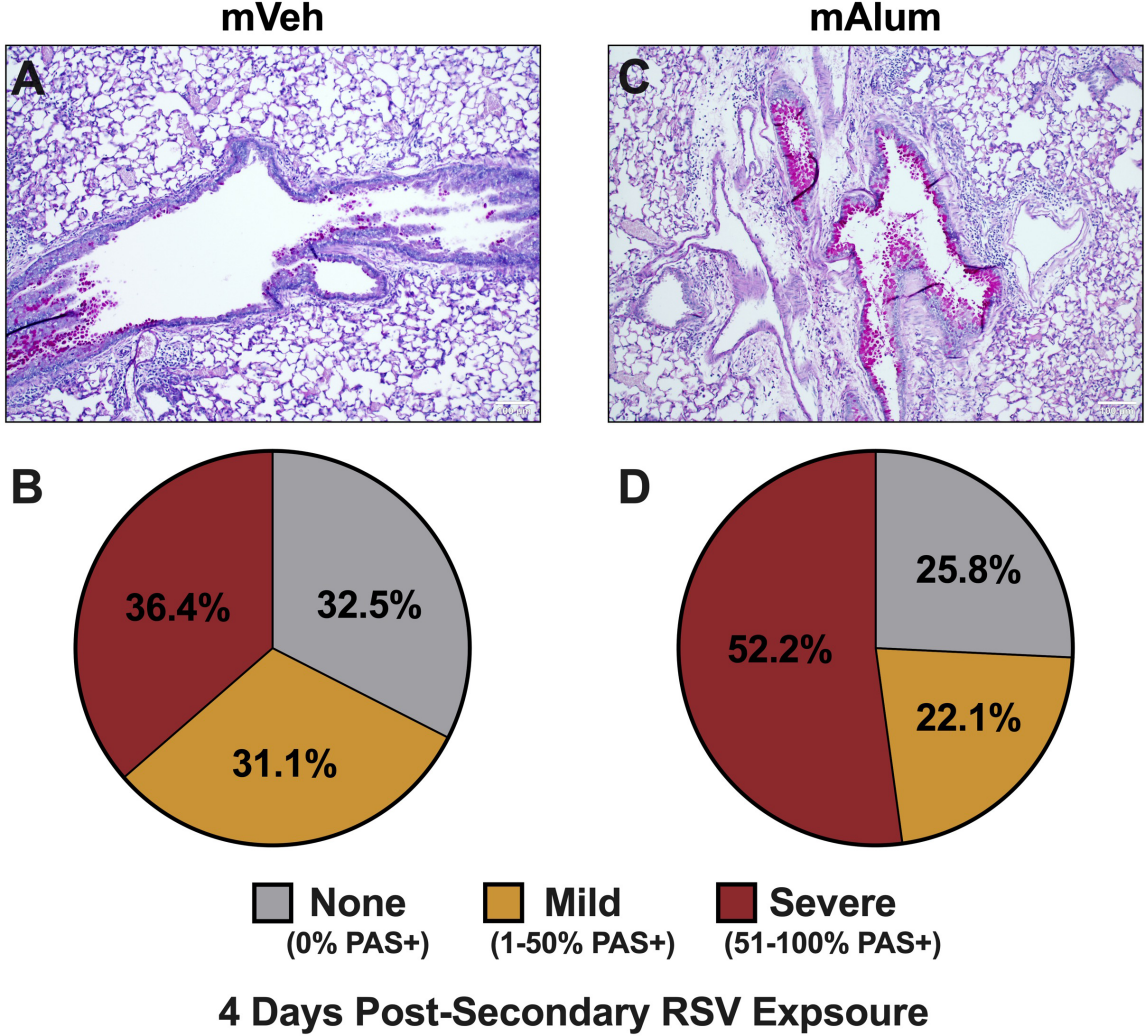
Mucus metaplasia increased in mAlum offspring following secondary RSV exposure. At 4dpse, lungs were formalin-fixed, paraffin-embedded, and processed for PAS staining. Each panel represents an individual mouse from each offspring group **(A, C)**. Lung sections were scored as described in the methods and represented as a proportion of airways scored as having no PAS staining (none), airways with 1-50% PAS staining (mild), and airways with 51-100% PAS staining (severe) **(B, D)**. n=3 mice per group.

To assess mucus metaplasia, a hallmark of RSV-mediated disease, formalin-fixed lungs were quantified for PAS staining (39). In our model, 67.5% of airways in mVeh offspring stained positively for mucin production, with 36.4% of those airways classified as severe after secondary RSV exposure (**Figures 5A, B** **)**. Comparatively, 74.3% of airways in mAlum offspring displayed mucus hyperproduction, with 52.2% of those scored as severe (**Figures 5C, D** **)**. Collectively, these results demonstrate the mAlum offspring have enhanced mucus metaplasia with an increased, albeit non-significant, severity of mucus production following RSV re-challenge. It is important to note that this increase in mucus occurred despite undetectable RSV replication during primary (**Figure 1C**) and secondary (**Figure 1D**) exposure.

### 3.6 Th2 responses are sustained while cytotoxic CD8^+^ T cells increase in mAlum offspring during late secondary RSV responses

**Figure 6:**
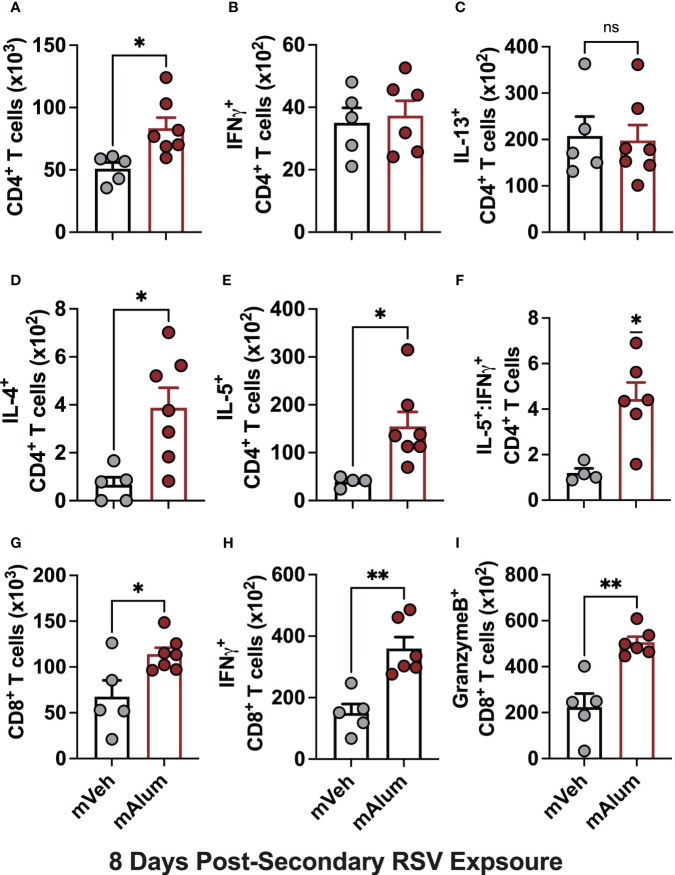
Th2 responses are sustained as cytotoxic CD8^+^ T cells increase in mAlum offspring. At 8dpse, total BAL CD4^+^ T cells **(A)**, as well as intracellular production of IFNγ, **(B)**, IL-13 **(C)**, IL-4 **(D)**, and IL-5 **(E)** by CD4^+^ T cells were quantified in mVeh and mAlum offspring, generated as described in **Figure 1**. From these totals, the ratio of IL-5:IFNγ CD4^+^ T cells was calculated **(F)**. Total BAL CD8^+^ T cells **(G)**, as well as those producing IFNγ **(H)**, and GranzymeB **(I)** were quantified. Data are represented as mean ± SEM (n=5-7 mice per group). Statistical significance was calculated using an unpaired t-test **(A-E, G-I)** or Wilcoxon signed-rank test **(F)**. ns – nonsignificant, *p ≤ 0.05 and **p ≤ 0.01.

To test if the robust T cell activation observed in mAlum offspring at 4dpse was an early, but quickly resolving anti-RSV response, we performed the same T cell analysis at 8dpse. Total CD4^+^ T cells were significantly higher in mAlum offspring compared to mVeh at 8dpsi (**Figure 6A**). While both IFNγ- and IL-13-producing CD4^+^ T cells increased 2-fold relative to the 4dpse timepoint (**Figures 2B, C**), there remained no significant difference in the number of IFNγ^+^ (**Figure 6B**) and IL-13^+^ (**Figure 6C**) CD4^+^ T cells between mVeh and mAlum offspring at 8dpse. Despite an increase in IL-4^+^ CD4^+^ T cells in mVeh offspring from 4 to 8 dpse and a corresponding decrease in mAlum offspring, IL-4^+^ CD4^+^ T cells remained significantly higher in mAlum compared to mVeh offspring at 8dpse (**Figure 6D**). IL-5^+^ CD4^+^ T cells increased dramatically in the airways of mVeh and mAlum offspring but remained significantly higher in mAlum compared to mVeh offspring (**Figure 6E**), maintaining the significantly higher IL-5^+^:IFNγ^+^ CD4^+^ T cell ratio in these offspring at 8dpse (**Figure 6F**).

While total CD8^+^ T cells and those producing IFNγ and GranzymeB were reduced in mAlum vs mVeh offspring at 4dpse (**Figures 2G–I**), they were markedly increased in mAlum vs. mVeh offspring by 8dpse (**Figures 6G–I**). Despite an increase in total CD8^+^ T cells in both mVeh and mAlum offspring between 4 and 8dpse, CD8^+^ T cells increased 6.4-fold in mAlum offspring vs 1.3-fold in mVeh offspring (**Table 1**). Similarly, IFNγ^+^ CD8^+^ T cells also increased in mVeh and mAlum offspring between 4 and 8 dpse (**Table 1**). Importantly, IFNγ-producing CD8^+^ T cells increased 6-fold vs 1.4-fold in mAlum vs. mVeh offspring, respectively, which led to significantly higher IFNγ^+^ CD8^+^ T cells in mAlum offspring at 8dpse (**Table 1**). Notably, CD8^+^ T cells producing GranzymeB increased 5.6-fold between 4 and 8dpse in mAlum offspring (**Table 1**). This increase, in combination with the concurrent decrease in GranzymeB^+^ CD8^+^ T cells in mVeh offspring between the same timepoints, led to significantly more GranzymeB^+^ CD8^+^ T cells in the mAlum offspring at 8dpse (**Figure 6I**). Taken together, these results demonstrate that, in mAlum offspring, the Th2-skewed response is sustained through 8dpse while the cytotoxic CD8^+^ T cell response increases, despite the absence of replicating RSV (**Figure 1D**).

**Table 1:** Fold change in cytotoxic CD8^+^ T cell populations between mVeh and mAlum offspring from 4 to 8 days post-secondary exposure.

CD8 T cell changes over time	mVeh	mAlum
CD8^+^ T cells	+1.3	+6.4
IFNy^+^ CD8^+^ T cells	+1.4	+6
GranzymeB^+^ CD8^+^ T cells	-1.7	+5.6

### 3.7 Severity of mucus metaplasia increases in mAlum offspring during late secondary RSV responses

**Figure 7:**
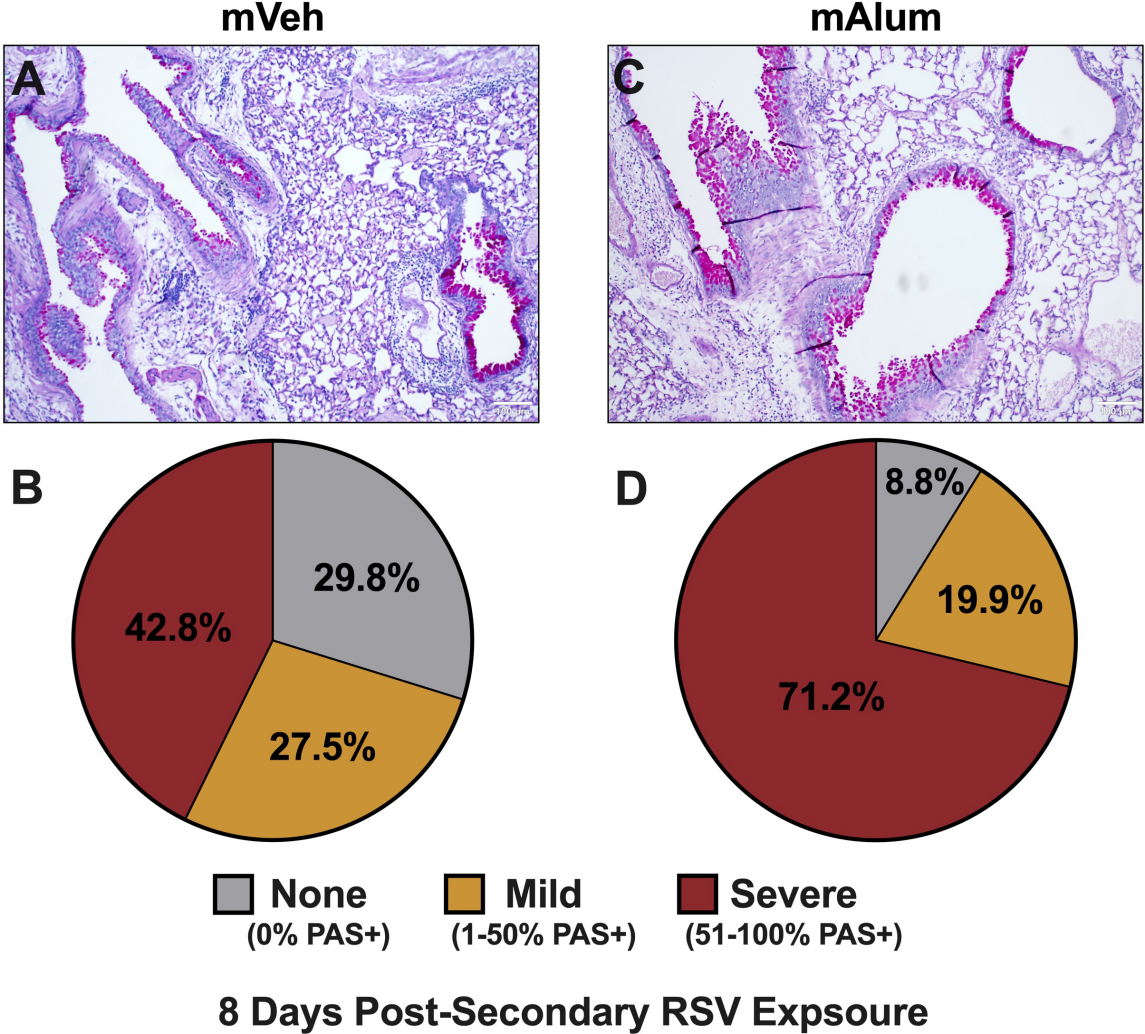
Worsening mucus production in mAlum offspring over course of secondary RSV exposure. At 8dpse, lungs were formalin-fixed, paraffin-embedded, and processed for PAS staining. Each panel represents an individual mouse from each offspring group **(A, C)**. Lung sections were scored, and the proportion of airways scored as having no PAS staining (none), airways with 1-50% PAS staining (mild), and airways with 51-100% PAS staining (severe) was calculated **(B, D)**. n=3 mice per group.

To assess the impact of sustained Th2 responses in combination with increased cytotoxic activity of CD8^+^ T cells on immunopathology, bronchovascular inflammation and mucus metaplasia were assessed in mVeh and mAlum offspring at 8dpse. We showed that total CD4^+^ (**Figure 6A**) and CD8^+^ T cells (**Figure 6G**) were increased in the lavage fluid harvested at 8dpse. However, no significant difference was observed in the frequency of bronchovascular bundles with inflammation in lungs harvested after lavage (**Supplementary Figures 6E-G**) and both groups displayed similar distributions in the severity of inflammation (**Supplementary Figure 6H**). In mVeh offspring, the percentage of airways staining positive for mucin modestly increased to 70.3% at 8dpse (from 67.5% at 4dpse), with those airways classified as severe increasing slightly to 42.8% at 8dpse (**Figures 7A, B**). Importantly, the percentage of airways scored as severe in mAlum offspring increased nearly 20% by 8dpse (52.2% at 4dpse vs. 71.2% at 8dpse). This marked increase in airway mucus severity resulted in over 90% of airways staining positive for mucin in mAlum offspring at 8dpse (**Figures 7C, D**). Collectively, these results demonstrate that both mVeh and mAlum offspring have persistent immunopathology, with the severity of mucus production increasing in mAlum offspring at 8 days post-secondary RSV exposure. Furthermore, the increased mucus production in mAlum offspring suggests that, in our model, maternal immunization with PreF+Alum does not mitigate mucus metaplasia following secondary RSV exposure.

### 3.8 CD4^+^ T cell depletion during secondary RSV exposure reduces lung pathology

**Figure 8:**
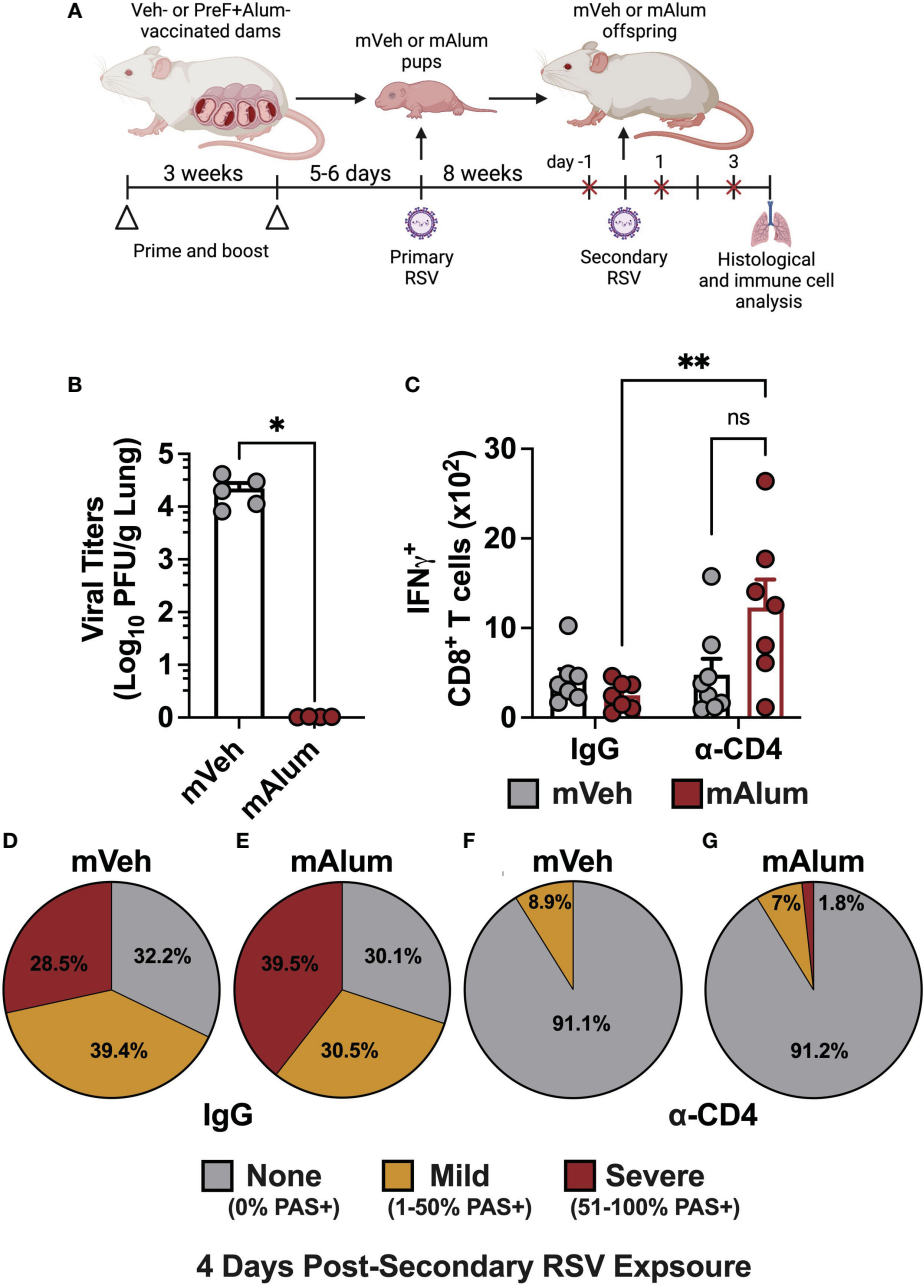
CD4^+^ T cells contribute to mucus production, but not viral control in mAlum offspring. Pregnant dams were vaccinated, and pups were primarily challenged with RSV and aged to adulthood as described in **Figure 1**. Adult mVeh and mAlum offspring were administered isotype (IgG) or CD4-depleting (α-CD4) antibody starting 1 day prior to secondary RSV challenge and every 2 days through sacrifice **(A)**. Left lungs were harvested for viral titer analysis **(B)** while IFNγ^+^ CD8^+^ T cells were quantified from the BAL **(C)**. The proportion of airways scored with no, mild, and severe PAS staining was calculated in IgG-treated mVeh **(D)** and mAlum **(E)** offspring, as well as CD4-depleted mVeh **(F)** and mAlum **(G)** offspring. Data are represented as mean ± SEM (n=5-8 mice per group). Statistical significance was calculated using an unpaired t-test **(B)** or two-way ANOVA with Sidak’s multiple comparison test **(C)**. *p ≤ 0.05 and **p ≤ 0.01.

The contribution of CD4^+^ T cells to lung pathology during secondary RSV challenge in mice primarily exposed as infants is well-documented (31, 40). To assess their contribution in our model of maternal RSV immunization, temporal, antibody-mediated depletion of CD4^+^ T cells was employed to eliminate CD4^+^ T cells immediately prior to secondary RSV challenge and through 4dpse. Briefly, mVeh and mAlum offspring were exposed to RSV as neonates and aged to adulthood, as described in **Figure 1**. One day prior to secondary RSV challenge and every other day through sacrifice at 4dpse, offspring were treated with isotype (IgG) or α-CD4 depleting antibodies (**Figure 8A**, red x). Treatment with α-CD4, but not IgG isotype, resulted in a significant and near-complete elimination of airway CD4^+^ T cells among live cells in the airway (**Supplementary Figure 7A**). Of note, while overall cell recovery in this experiment was low, it did not impact the statistical significance between α-CD4-treated and IgG isotype controls. Unlike mVeh offspring, mAlum offspring treated with α-CD4 had undetectable levels of replicating virus (**Figure 8B**), suggesting that CD4^+^ T cells were dispensable for the control of viral replication in these offspring at 4dpse. This finding prompted the investigation of IFNγ^+^ CD8^+^ T cells. Consistent with our earlier findings, IFNγ-producing CD8^+^ T cells trended lower in the IgG isotype-treated mAlum vs. mVeh offspring (**Figure 8C**). Though no difference was observed in total IFNγ^+^ CD8^+^ T cells between IgG isotype- and α-CD4-treated mVeh offspring, there were significantly more IFNγ-producing CD8^+^ T cells in α-CD4-treated compared to IgG isotype-treated mAlum offspring that likely contributed to the control of viral replication in α-CD4-treated mAlum offspring (Figure 8B). Consistent with our earlier findings (**Figure 5**), 67.9% of airways stained positively for mucin in IgG isotype-treated mVeh offspring at 4dpse, with 28.5% of those airways considered severe (**Figure 8D** **;** **Supplementary Figure 7B**). In IgG isotype-treated mAlum offspring, 70% of airways stained positively for mucin at 4dpse (**Figure 8E**, **Supplementary Figure 7C**). Importantly, 39.5% of airways were scored as severe (**Figure 8E**), which is consistent with the trend toward increased severity in mAlum offspring at 4dpse shown earlier (**Figure 5**). Conversely, airway mucus production was reduced ~8-fold in both mVeh and mAlum offspring after treatment with α-CD4, resulting in a near complete amelioration of airway mucus metaplasia following secondary RSV exposure (**Figures 8F, G**, **Supplementary Figures 7D, E**). Collectively, these results demonstrate a central role for CD4^+^ T cells in the development of airway pathology following secondary RSV exposure in both mVeh and mAlum offspring.

### 3.9 hILC2s are reduced in the absence of CD4^+^ T cells following secondary RSV exposure

**Figure 9:**
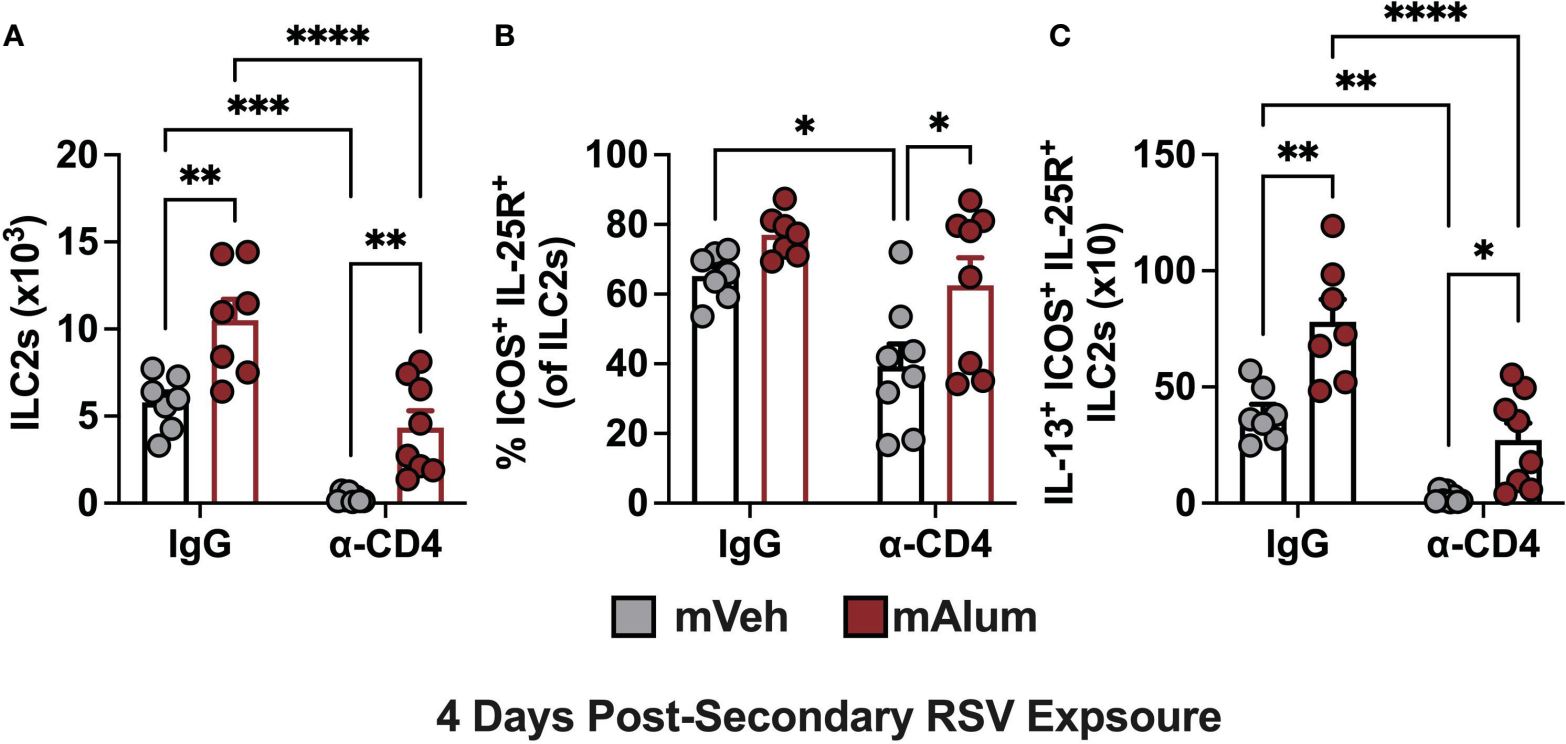
Hyperresponsive ILC2s are reduced in the absence of CD4^+^ T cells. Adult mVeh and mAlum offspring were treated as described in **Figure 8**. Total lung ILC2s **(A)**, frequency of lung ILC2s dual positive for ICOS and IL-25 **(B)**, and IL-13^+^ ICOS^+^ IL-25R^+^ ILC2s **(C)** were quantified from IgG- and α-CD4-treated mVeh and mAlum offspring. Data are represented as mean ± SEM (n=7-8 mice per group). Statistical significance was calculated using two-way ANOVA with Sidak’s multiple comparison test **(C)**. *p ≤ 0.05 **p ≤ 0.01, ***p ≤ 0.001 and ****p ≤ 0.0001.

Previous work has demonstrated that crosstalk between CD4^+^ T cells and ILC2s leads to their mutual maintenance, expansion, and cytokine production and plays an important role in the etiology of several lung diseases (41, 42). Specifically, depletion of CD4^+^ T cells during RSV infection leads to reduced ILC2 totals, indicating that CD4^+^ T cells contribute to RSV-mediated ILC2 activation, with their collective interaction exacerbating disease (42). We hypothesized that with CD4^+^ T cell depletion, the ILC2 population measured following secondary RSV challenge would also be reduced. ILC2s were assessed in mVeh and mAlum offspring following CD4^+^ T cell depletion at 4dpse, as described in **Figure 8A**. As expected, depletion of CD4s (α-CD4) significantly reduced ILC2s in both mVeh and mAlum offspring (**Figure 9A**). Similar to mice with CD4^+^ T cells (**Figure 3**), mAlum offspring had significantly more ILC2s compared to mVeh in the absence of CD4^+^ T cells (**Figure 9A**). When the ILC2 population was further characterized for expression of hyperresponsive markers - ICOS and IL-25R - we observed a slight increase in the frequency of ICOS^+^ IL-25R^+^ hILC2s in IgG isotype-treated mAlum offspring compared to mVeh offspring (**Figure 9B**, IgG). Importantly, while the frequency of ICOS^+^ IL-25R^+^ hILC2s was significantly reduced in mVeh offspring following CD4^+^ T cell depletion, there was no reduction in the frequency of hILC2s in mAlum offspring (**Figure 9B**). IL-13^+^ ICOS^+^ IL-25R^+^ hILC2s were significantly increased in IgG isotype-treated mAlum offspring compared to mVeh offspring (**Figure 9C**). While depletion of CD4^+^ T cells significantly reduced the number of IL-13-producing ICOS^+^ IL-25R^+^ hILC2s in both offspring groups, mAlum offspring had significantly more IL-13^+^ ICOS^+^ IL-25R^+^ hILC2s relative to mVeh offspring following secondary RSV exposure in the absence of CD4^+^ T cells (**Figure 9C**) and in the absence of replicating virus (**Figure 8B**). Taken together, this data confirms that depletion of CD4^+^ T cells significantly alters the ILC2 population following secondary RSV exposure, but the enhanced ILC2 response in mAlum vs. mVeh offspring persists.

## 4 Discussion

Maternal RSV vaccination has emerged as a promising approach to provide much-needed, early-life protection from severe RSV infection and associated hospitalization (10). While maternal RSV vaccination has demonstrated efficacy in reducing medically significant RSV lower respiratory tract infections during infancy, the subsequent changes in immune response to repeated RSV exposure are largely unknown (9). Furthermore, the WHO Working Group on Respiratory Syncytial Virus Vaccination During Pregnancy has concluded that current clinical efficacy trials of maternal RSV vaccine candidates are unlikely to demonstrate a significant effect on preventing RSV-associated disease sequelae due to limited sample sizes, highlighting the need for alternative study designs to estimate the impact of maternal RSV vaccine programs on disease sequelae (43). Toward this goal, our lab has established a clinically relevant murine model of maternal RSV vaccination and repeated RSV exposure. Using this novel model, we have demonstrated that the presence of highly neutralizing preF-specific matAb during infant RSV exposure protects from active RSV infection but facilitates the enhancement of type-2 immunity on secondary RSV challenge and is associated with exacerbated RSV-mediated airway disease.

Previous work has shown that IL-33, an alarmin released following tissue damage, is a potent sensitizing signal that leads to the establishment of a long-lived, lung-resident ILC2 population capable of exaggerated responses following exposure to the same and unrelated antigens (37). RSV infection is known to elicit IL-33, along with associated ILC2 expansion and activation following virus-induced airway epithelial damage, a response that is more potent in neonates compared to adults (34). Thus, it was expected that mVeh offspring, which had replicating RSV in their lungs during primary neonatal infection, would establish a hILC2 population capable of contributing to severe RSV immunopathogenesis upon re-exposure. This hypothesis was consistent with data from several studies showing that early-life infection with several respiratory pathogens leads to exaggerated ILC2 responses upon re-exposure to the same pathogen (36, 44). In a neonatal mouse model of intranasal rhinovirus (RV) infection, exaggerated ILC2 expansion and mucus metaplasia was observed following heterologous RV infection (44). In a model of secondary RSV infection, mice infected with RSV for the first time as neonates had exaggerated ILC2 cytokine responses combined with enhanced airway inflammation upon secondary adult RSV infection (36). Collectively, these results demonstrate that viral-mediated activation of ILC2s during infancy leads to the establishment of hILC2s capable of contributing to the immunopathogenesis associated with secondary RSV infection.

Unexpectedly, mAlum offspring, which were completely protected from replicating virus during primary neonatal RSV exposure, established a robust, activated hILC2 population that exceeded the hILC2 population in mVeh offspring upon RSV re-exposure. Though the mechanism has not been fully elucidated, the presence of hILC2s in mAlum offspring suggests that ILC2s were activated during primary neonatal RSV exposure when virus was completely neutralized in the presence of matAb : RSV immune complexes (ICs). As the neonatal lungs mature, ILC2s increase (45), resident alveolar macrophages adopt an alternatively activated phenotype (18, 46), and CD11b^+^ DCs, known for promoting Th2 responses (45) are enriched, altogether fostering a predominant type-2 immune cells bias within the lungs of neonates (47). Moreover, binding of ICs comprised of IgG1, the predominant immunoglobulin subclass generated following vaccination with alum adjuvant (48), polarize M2 macrophages, that subsequently activate ILC2s and Th2 CD4^+^ T cells to further promote type-2 immune responses (49–52). Therefore, it is plausible that mAlum pups, who have a high IgG1/IgG2a ratio (**Supplementary Figure C**), are exposed to high levels of IgG1 ICs during early-life RSV exposure, leading to M2 macrophage polarization and activation of type-2 immune cell populations that are re-activated following subsequent exposure to RSV. Another possible mechanism of early-life ILC2 activation in the presence of neutralizing matAb : RSV ICs is through activation of C3aR-expressing ILC2s. Gour and colleagues demonstrated that ILC2s can respond directly to C3a via C3aR ligation, inducing IL-13 expression and enhancing ILC2-mediated Th2 polarization (53). The unexpected results in mAlum offspring following secondary RSV exposure highlight our incomplete understanding of early-life ILC2 activation and necessitate additional studies that elucidate the mechanism of neonatal ILC2 activation in the context of maternal RSV immunization at both primary RSV challenge and subsequent viral exposures.

Early life RSV infection is associated with the subsequent development of wheezing and asthma (2, 3, 54). Though no causal link has been reported, ILC2s are potent contributors to allergic asthma so it is intriguing to consider that hILC2s may play a role in RSV-associated allergic asthma and wheezing. Despite a persistent elevation of IL-13^+^ hILC2s in mAlum versus mVeh groups, IL-13^+^ hILC2s were reduced in both groups following CD4^+^ T cell depletion and was associated with a marked reduction in mucus metaplasia in both groups. These findings are consistent with previously published results showing that depletion of Th2 cells during RSV infection inhibits ILC2 activation and secretion of type 2 cytokines, leading to a reduction in disease severity (42, 55). However, the parallel decrease in ILC2s in the absence of CD4^+^ T cells confounds the direct contribution of CD4^+^ T cells versus their maintenance, expansion, and activation of ILC2s to the observed immunopathology in our model. Untangling the distinct contribution of these cells to the enhanced immunopathology in our model is the focus of future work. A possible explanation for the reduction in mucus in the mAlum group is the parallel increase in IFNγ-producing CD8^+^ T cells in the mAlum group (**Figure 8C**). Mitchell et al. and others have shown that IFNγ can counteract the mucus-driving potential of IL-13 and may explain the reduced mucus metaplasia in the mAlum group despite persistent IL-13^+^ hILC2s (56–60). Furthermore, the comparative persistence and activation of hILC2s in CD4-depleted mAlum vs. mVeh offspring (**Figure 9C**) suggests the hILC2 populations in these offspring possess cell-intrinsic differences that alter their survival, responsiveness, and/or activity upon secondary activation; an observation that must be confirmed through epigenetic analysis. Steer and colleagues have demonstrated that IL-33-mediated activation of ILC2s during the neonatal period leads to a more functionally competent ILC2 population in adulthood (61). This work, however, did not assess whether neonatal ILC2s activated under different conditions leads to the development of functionally distinct hILC2s. Ultimately, our unexpected findings suggest that ILC2s activated during primary RSV infection in the presence of matAb may develop into an hILC2 population capable of contributing to enhanced airway disease following secondary RSV exposure, a mechanism that warrants further investigation.

Maternal antibody, though largely protective against most pathogens, remains controversial in the context of early-life RSV infection. While high levels of matAbs have been correlated with protection from severe early-life RSV disease and hospitalization, most cases of severe disease occur in infants less than 6 months of age, when matAbs are highest (1, 7–9). This led some to suggest that matAbs derived from natural RSV infection, which sub-optimally neutralize RSV, may worsen disease (62). This concern was largely mitigated by the discovery and subsequent stabilization of the RSV pre-fusion F protein (preF). When used as a vaccine antigen, preF elicits high levels of RSV-neutralizing antibodies, making it an ideal vaccine antigen for maternal RSV vaccination approaches (27, 63). Though the prevailing assumption is that transfer of highly neutralizing RSV-specific matAbs will provide immediate and complete protection from severe early-life RSV infection, few studies directly assess their influence on how it may alter long-term infant immunity. Furthermore, studies investigating the effects of RSV-neutralizing monoclonal antibodies (mAb) and matAbs on secondary RSV infection and associated disease sequelae are also sparse and have reported mixed results (64–68). While prevention of early-life RSV infection by palivizumab, an RSV-neutralizing monoclonal antibody, was shown to have a protective role against recurrent wheeze, use of the high-affinity anti-RSV monoclonal antibody, motavizumab, had no effects on rates of medically attended wheeze, despite prevention of severe early-life RSV infection (64–67). Intriguingly, one study utilizing data from the Danish National Birth Cohort concluded that high levels of RSV-neutralizing matAbs, resulting from natural infection, in the first 6 months of life was associated with an increased risk of developing recurrent wheeze in later childhood (68).

While our results align with the latter studies and suggest that RSV-neutralizing matAb may enhance type-2 immune responses following secondary RSV exposure, the diverse function and complex biology of IgGs and their cognate receptors (FcγRs) leave room for vaccine modifications that may improve outcomes (69). High levels of IgG, such as those elicited following vaccination with preF+Alum, results in the generation of smaller ICs, leading to suboptimal crosslinking of FcγRs and alteration of downstream effector function (70, 71). Thus, careful consideration must be given to the amount of RSV-neutralizing matAb transferred to offspring. Another important consideration is the glycosylation pattern of the Fc region of IgGs, which can profoundly alter their affinity and stability (72–74). Specifically, the presence, extent, and type of Fc glycosylation alters the affinity of IgGs for activating vs. inhibitory FcγRs, shifting the combinatorial engagement of activating vs. inhibitory FcγRs and impacting downstream cellular responses to ICs (69, 75–77). It would therefore be sensible to leverage the various matAb IgG modifications to optimally crosslink FcγRs and fine-tune infant immune responses. Lastly, previous work in our lab has demonstrated that pre-existing immunity to RSV in PreF+Alum-vaccinated dams alters the subclass of IgG in favor of higher IgG2a levels (23). The studies presented herein used RSV-naïve dams, which resulted in the predominant transfer of IgG1 to resulting mAlum offspring (**Supplementary Figure 4C**). It remains unknown if use of RSV pre-immune dams, and by extension, passive transfer of more IgG2a, would shift the Th1/Th2 balance in resulting offspring and improve secondary RSV outcomes associated with maternal RSV vaccination. Additionally, use of different vaccine adjuvants could be leveraged to alter the resulting IgG subclass to further improve secondary RSV outcomes (78). Viral neutralization is considered the gold standard for vaccine efficacy, leading most studies, including ours, to prioritize neutralizing antibody titers. However, focus must also be given to IgG subtype, Fc modifications, and quantity of matAb transferred to offspring.

Ultimately, the studies presented here demonstrate that highly neutralizing preF-specific matAbs contribute to active anti-RSV immunity in far greater ways than simply neutralizing virus and have a marked influence on primary and secondary RSV immune responses. The knowledge gap in this area underscores the need for additional studies that determine the optimal matAb quantity and qualities that balance protection vs. pathogenesis to better inform the rationale design of maternal RSV vaccine candidates.

## Data availability statement

The raw data supporting the conclusions of this article will be made available by the authors, without undue reservation.

## Ethics statement

The animal study was reviewed and approved by University of Pittsburgh Institutional Animal Care and Use Committee.

## Author contributions

JK, KEm, and KEi contributed to the overall study design, execution, and interpretation of data and writing of the paper. ML, MY, SG, and DB contributed to data generation and analysis. TP contributed to the analysis and interpretation of data. All authors contributed to the article and approved the submitted version.
