# Supplementary material for: Exacerbated lung inflammation following secondary RSV exposure is CD4+ T cell-dependent and is not mitigated in infant BALB/c mice born to PreF-vaccinated dams
Source: Front Immunol. 2023 Aug 14;14:1206026. doi: 10.3389/fimmu.2023.1206026 (PMC10461110; doi:10.3389/fimmu.2023.1206026)
Supplement: Supplementary file 1 [file DataSheet_1.pdf]

Supplementary Material

Type 2 inflammation following secondary RSV exposure of infants born to preF-vaccinated dams is $CD4^+$ T cell-dependent

Jessica L Kosanovich, Katherine M Eichinger, Madeline A Lipp, Sonal V Gidwani, Devarshi Brahmbhatt, Mark A Yondola, Timothy N Perkins, Kerry M Empey*

*** Correspondence:** Kerry M Empey: kme33@pitt.edu

1.0 Supplementary Figures

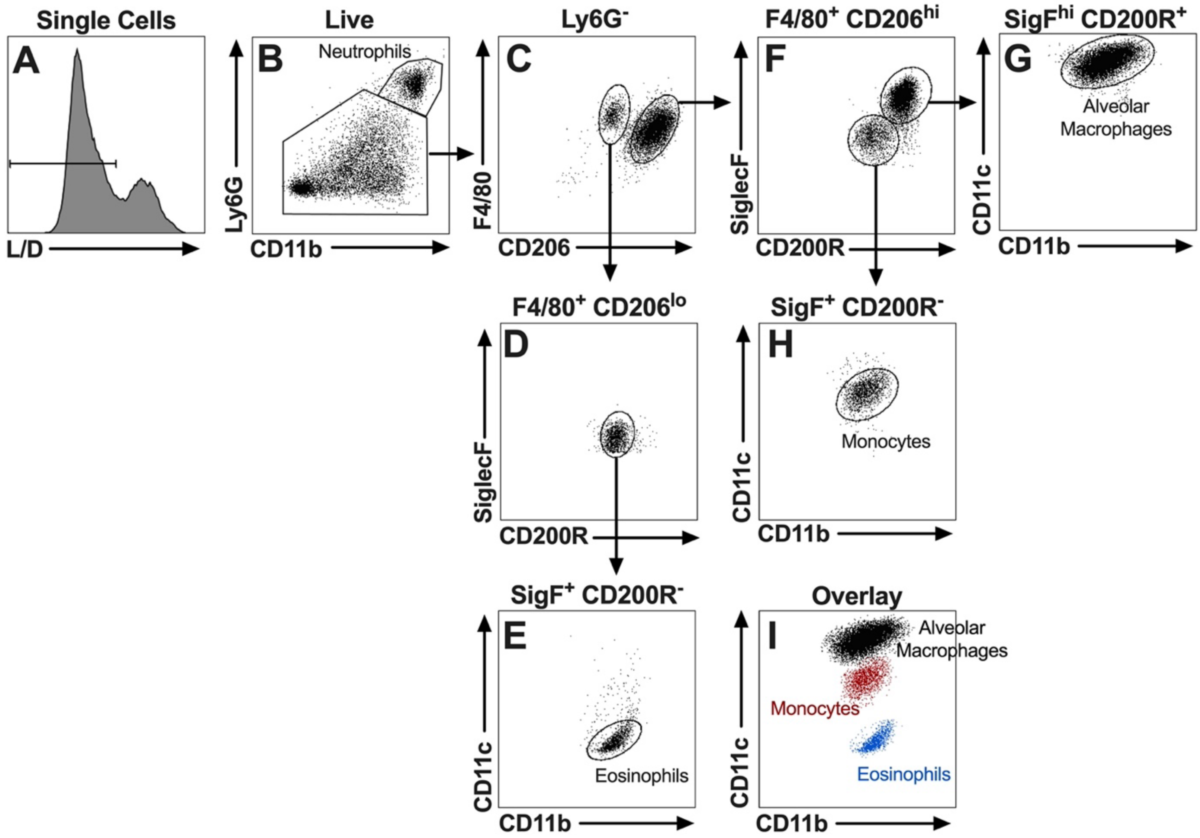

Supplementary Figure 1. Innate cell gating strategy. Following FSC vs. SSC gating to discriminate large cells in the BAL, aggregate exclusion (FSC-H vs FSC-W) was performed to isolate single cells, which were then analyzed for Live/Dead exclusion (L/D; A). L/D^- cells were then analyzed for CD11b vs. Ly6G expression (B). $CD11b^+$ $Ly6G^+$ were considered neutrophils, while $CD11b^{+/-}$ $Ly6G^-$ cells were further analyzed for the expression of F4/80 vs. CD206 (C). $F4/80^+$ $CD20^{lo}$ cells were then separated based on their expression of SiglecF and CD200R (D). $SiglecF^+$ $CD200R^-$ cells were then discriminated based on their expression of CD11c vs. CD11b, with $CD11c^-$ $CD11b^+$ cells considered eosinophils (E). $F4/80^+$ $CD206^{hi}$ cells were further discriminated based on their expression of SiglecF and CD200R (F). $SigF^{hi}$ $CD200R^+$ cells were then discriminated based on CD11c vs. CD11b expression, with $CD11c^+$ $CD11b^{+/-}$ considered alveolar macrophages (G). $SigF^+$ $CD200R^-$ cells were also further discriminated on their expression of CD11c vs. CD11b, with $CD11c^{lo}$ $CD11b^+$ cells considered monocytes (H). An overlay of eosinophils (E), alveolar macrophages (G), and monocytes (H) shows the distribution of these cell populations when assessed for CD11c vs CD11b expression (I).

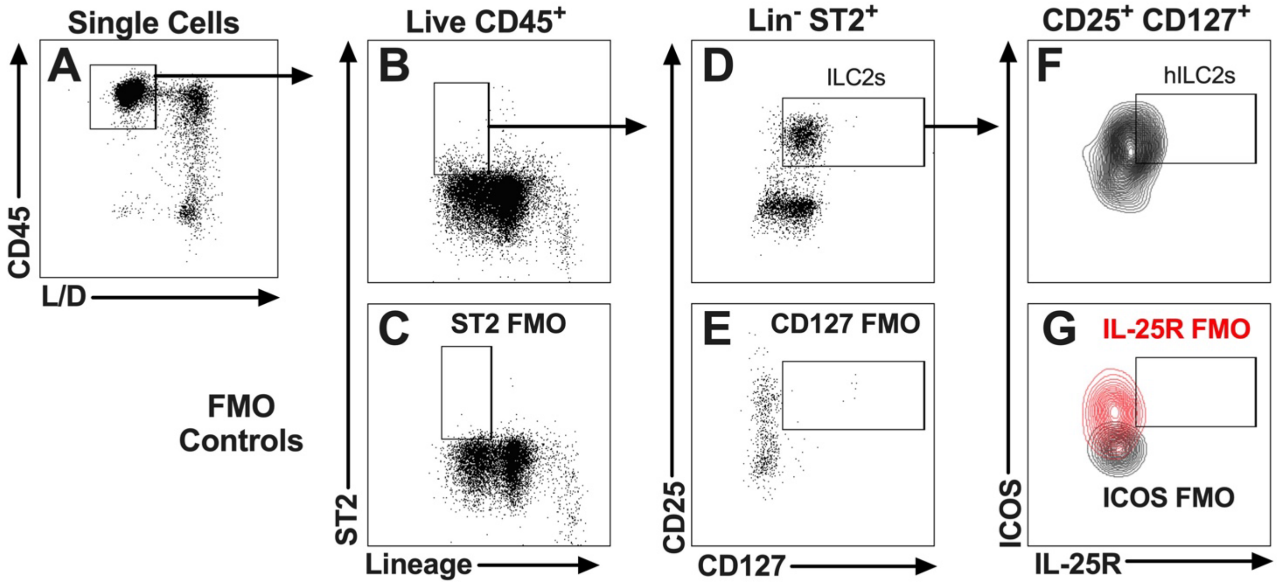

Supplementary Figure 2. ILC2 gating strategy. Following FSC vs. SSC gating to discriminate lymphocytes in the lung, aggregate exclusion (FSC-H vs FSC-W) was performed to isolate single cells, which were then analyzed for CD45 vs. Live/Dead exclusion (A). Live $CD45^+$ cells were then discriminated based on their expression of ST2 vs Lineage (Lin; B), using an ST2 FMO to determine ST2 positivity (C). Lin^- $ST2^+$ were then analyzed for CD25 vs CD127 expression (D), with a CD127 FMO used to identify CD127 positivity (E). $CD25^+$ $CD127^+$ cells were considered ILC2s. To identify hyperresponsive ILC2s (hILC2s), $CD25^+$ $CD127^+$ ILC2s were further assessed for ICOS and IL-25R expression (G), with $ICOS^+$ IL-$25R^+$ cells considered hILC2s. FMOs for ICOS and IL-25R were used to determine their respective positivity (G).

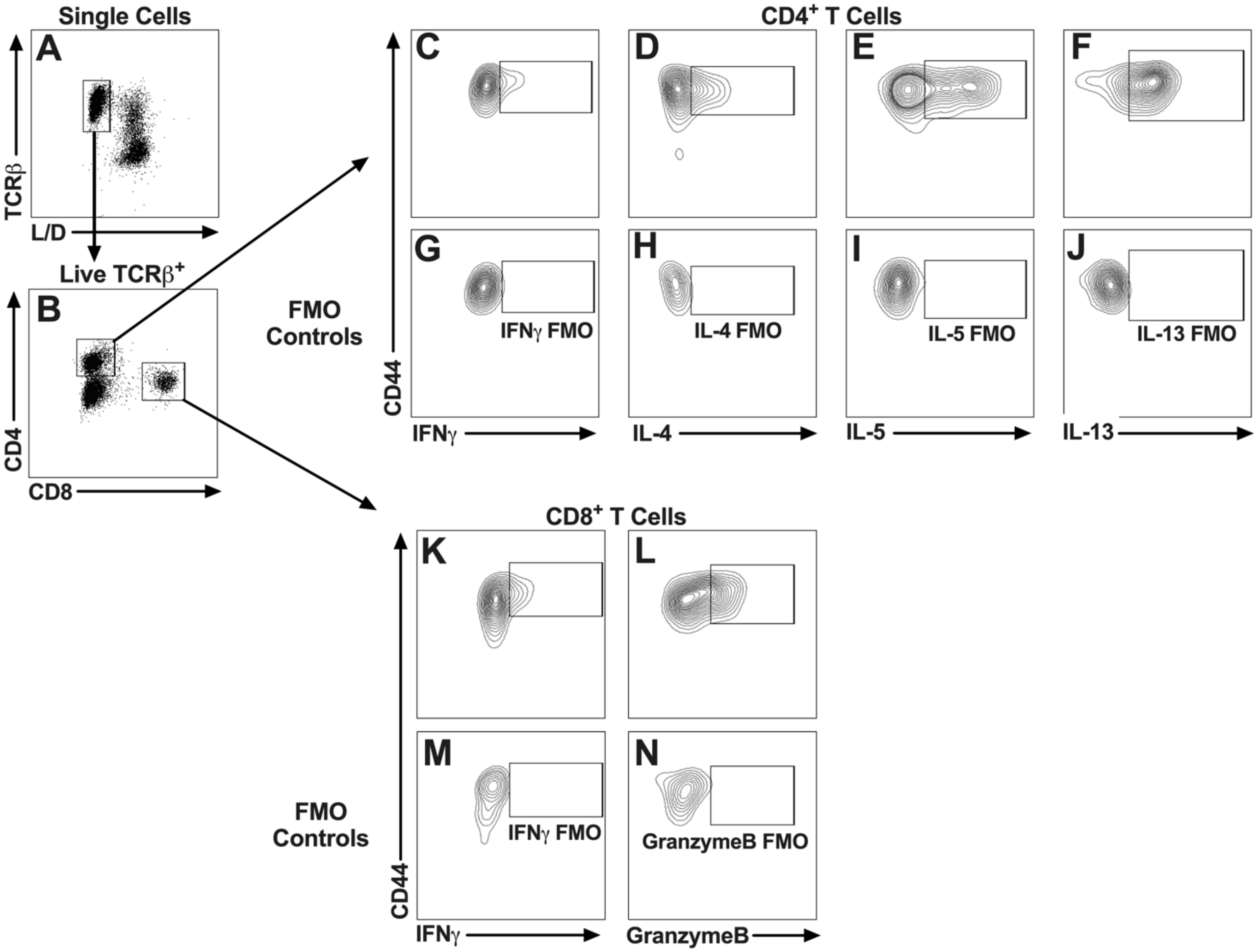

Supplementary Figure 3. T cell gating strategy. Following size discrimination and aggregate exclusion (as described in Supp Fig 1 and 2), single cells were assessed for TCRβ vs. Live/Dead expression (L/D; A). Live $TCR\beta^{+}$ cells were then further delineated based on their CD4 vs CD8 expression (B). $CD44^{+}$ $CD4^{+}$ T cells were then assessed for IFNγ (C), IL-4 (D), IL-5 (E), and IL-13 (F) expression, with FMOs used to determine the positive signal of each cytokine (G-J). CD44+ CD8+ T cells were analyzed for their expression of IFNγ (K) and GranzymeB (L), using FMOs to determine positivity (M-N).

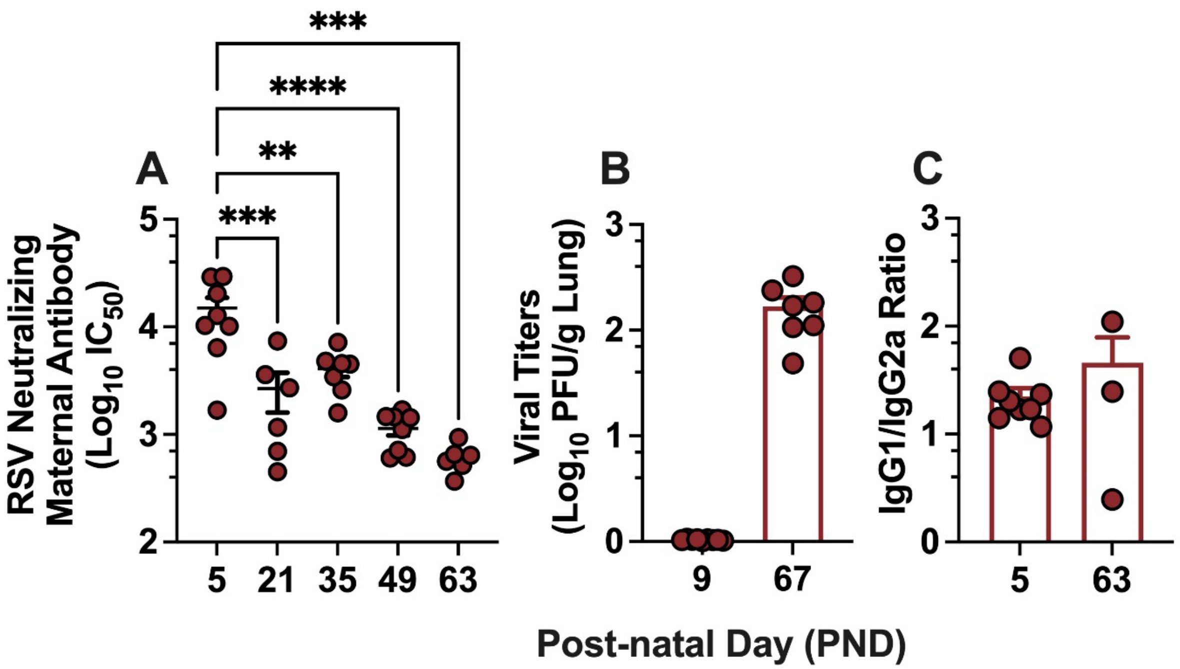

Supplementary Figure 4. Declining maternal RSV neutralizing antibody does not afford complete protection. Pre-challenge serum from mAlum offspring, generated as described in Figure 1, was analyzed for presence of RSV neutralizing maternal antibody at indicated timepoints as described in the methods (A). Viral titers were obtained from the left lungs of mAlum offspring following primary challenge at PND9 and PND67 (B). The ratio of RSV-specific IgG1 to IgG2a was calculated from pre-challenge serum at PND5 and PND63 in mAlum offspring (C). Statistical significance was calculated using an ordinary one-way ANOVA with Tukey's multiple comparison test (A) between timepoints. $^{**}p \le 0.01$, $^{***}p \le 0.001$ and $^{****}p \le 0.0001$.

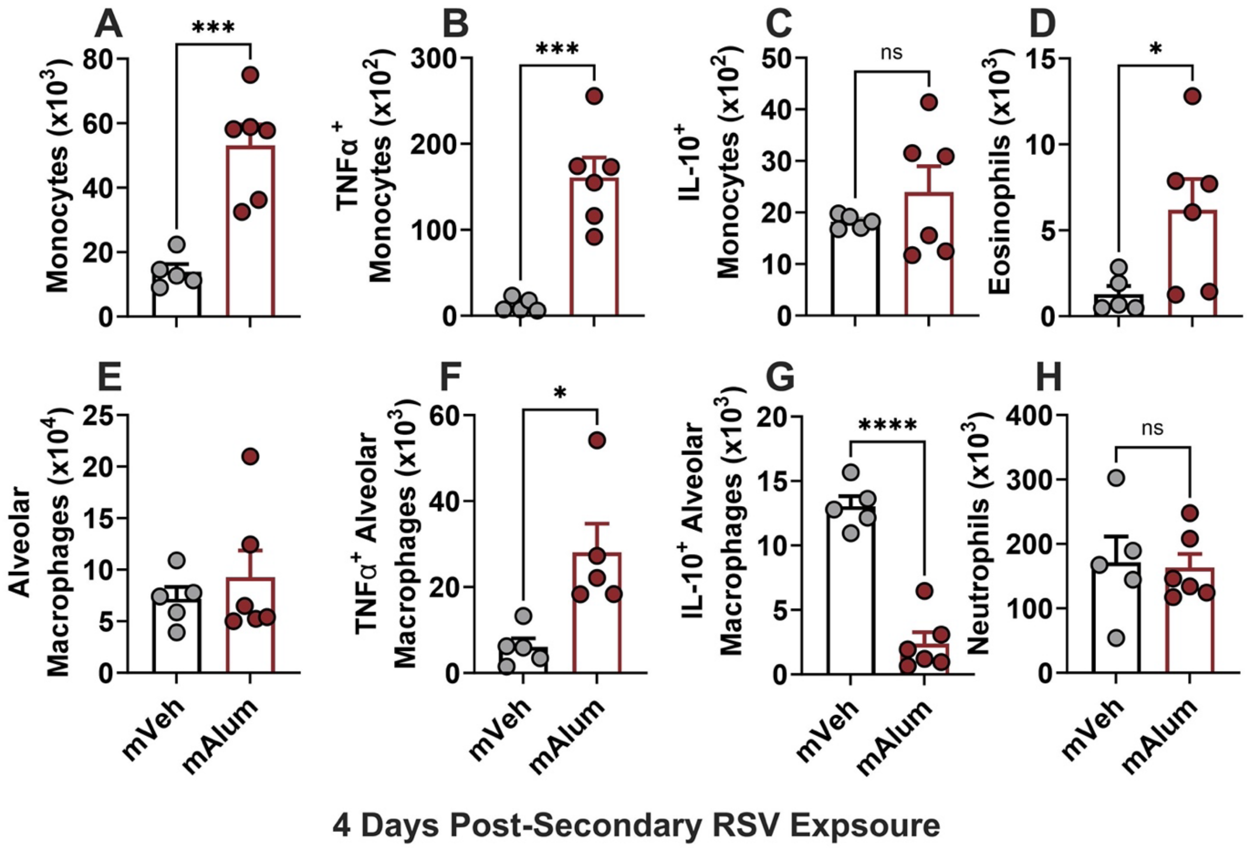

Supplementary Figure 5. Increased inflammatory macrophages and monocytes in mAlum offspring following secondary RSV exposure. mVeh and mAlum offspring were generated and exposed to RSV as described in Figure 1. At 4 days-post secondary exposure, total monocytes (A), along with those producing TNFα (B) and IL-10 (C) were quantified in the BAL. Eosinophils (D), as well as total alveolar macrophages (E), TNFα⁺ alveolar macrophages (F), IL-10⁺ alveolar macrophages (G), and neutrophils (H) were also quantified in the BAL. Data are represented as mean ± SEM (n=5-6 mice per group). Statistical significance was calculated using an unpaired t-test. ns – non-significant, *p ≤ 0.05, ***p ≤ 0.001 and ****p ≤ 0.0001.

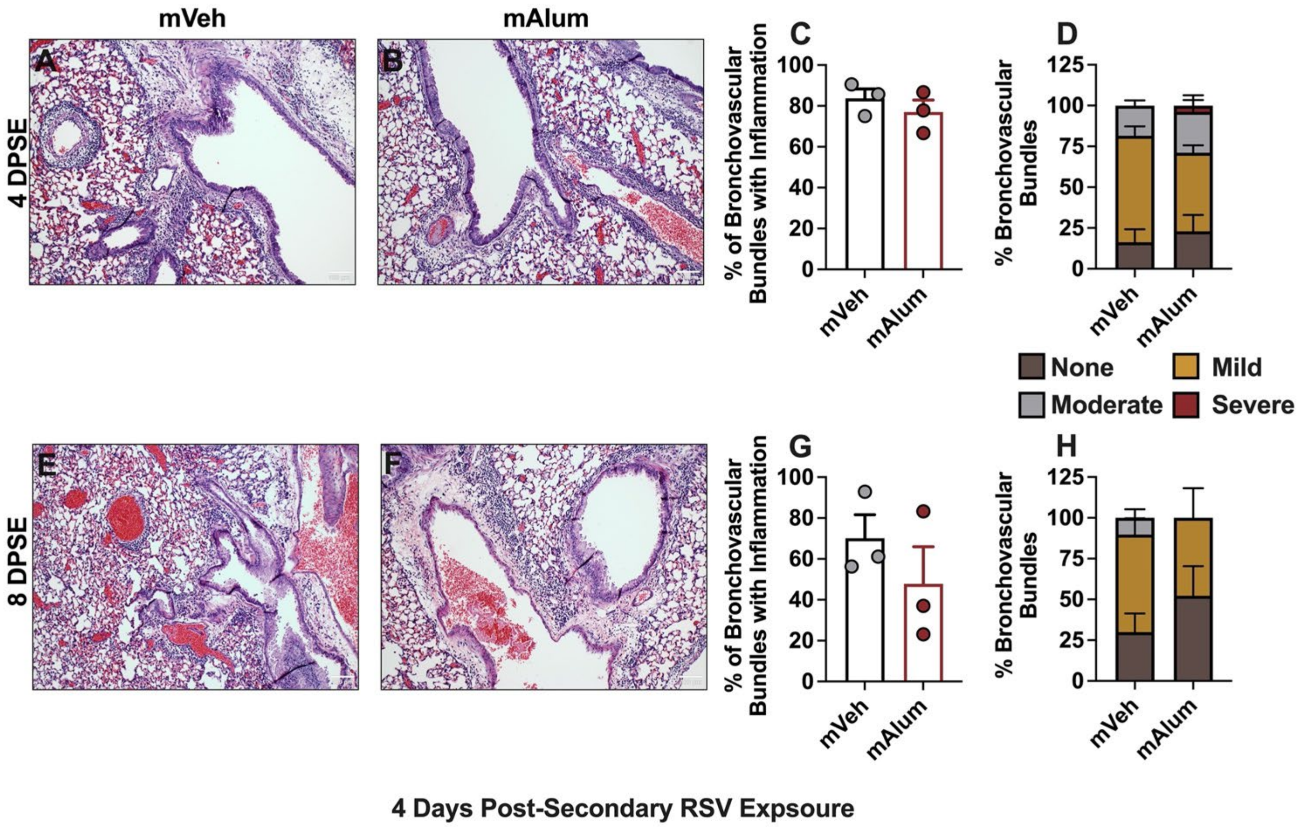

Supplementary Figure 6. Pulmonary inflammation is similar between mVeh and m Alum offspring following secondary RSV exposure. mVeh and mAlum offspring were generated and exposed to RSV as described in Figure 1. At 4- (A-D) and 8-days (E-G) post-secondary exposure, left lung sections were stained with hematoxylin and eosin (A-B, E-F). Each represents an individual mouse from the indicated group. Lung sections were scored by blinded pathologist and represented as the frequency of bronchovascular bundles with inflammation (C, G), in addition to the percentage of bronchovascular bundles scored as having no, mild, moderate, or severe inflammation (D, H). Statistical significance was calculated using an unpaired t-test (C-D, G-H).

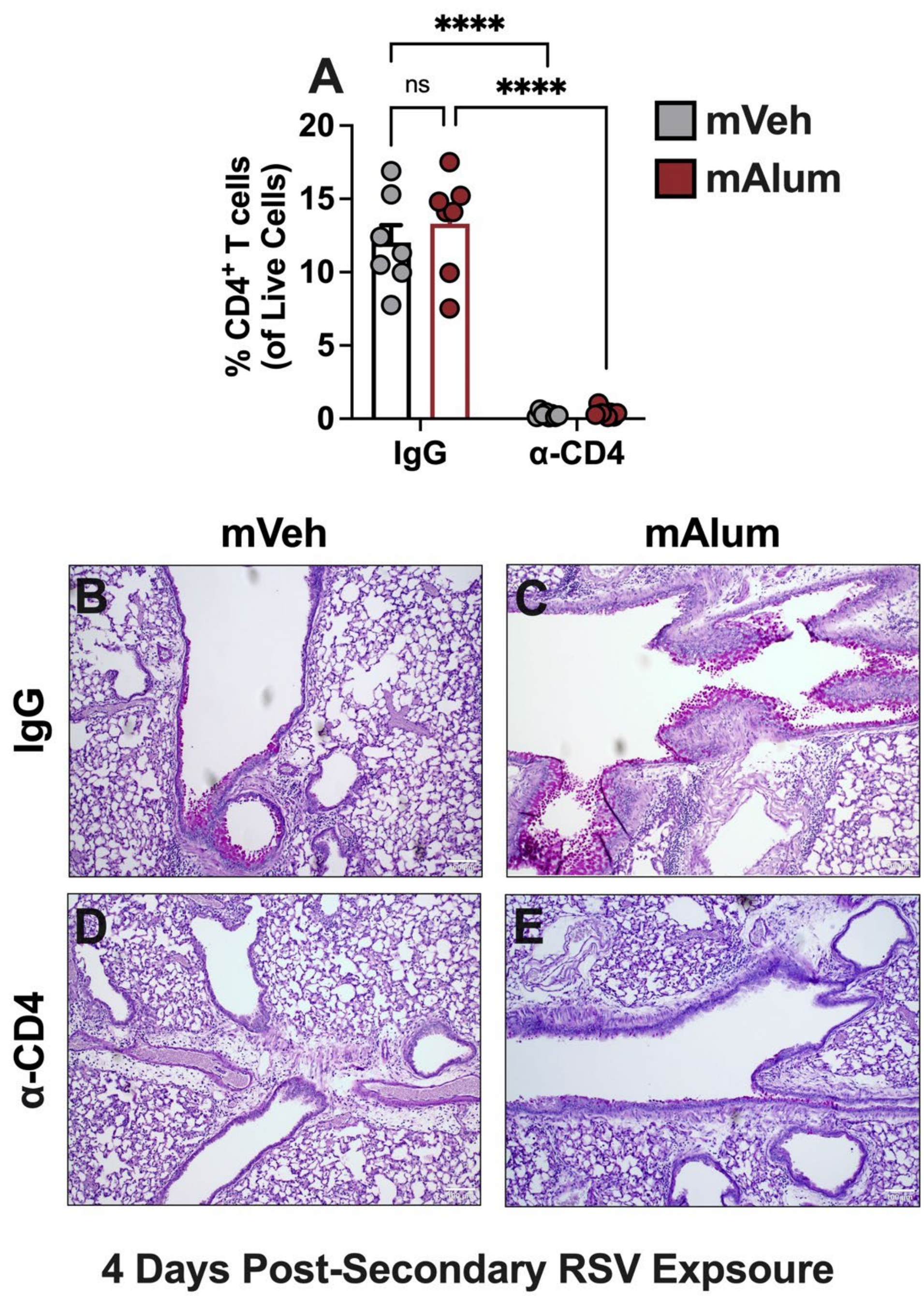

Supplementary Figure 7. Reduction of CD4⁺ T cells leads to absence of mucus production. mVeh and mAlum offspring were generated, exposed to RSV, and treated with IgG or α-CD4 as described in Figure 8. Frequency of $CD4^+$ T cells were quantified in the BAL (A). Representative PAS-stained lung sections from IgG-treated (B-C) and α-CD4-treated (D-E) mVeh and mAlum offspring. Data are represented as mean ± SEM (n=7-8 mice per group). Statistical significance was determined using a two-way ANOVA with Sidak's multiple comparison test. ns - non-significant, **p ≤ 0.01 and ****p ≤ 0.0001.
